# Splicing-accessible coding 3′UTRs control protein stability and interaction networks

**DOI:** 10.1186/s13059-020-02102-3

**Published:** 2020-07-29

**Authors:** Marco Preussner, Qingsong Gao, Eliot Morrison, Olga Herdt, Florian Finkernagel, Michael Schumann, Eberhard Krause, Christian Freund, Wei Chen, Florian Heyd

**Affiliations:** 1 Institute of Chemistry and Biochemistry, Freie Universität Berlin, Laboratory of RNA Biochemistry, Takustrasse 6, 14195 Berlin, Germany; 2 Berlin Institute for Medical Systems Biology, Max Delbrück Center for Molecular Medicine, Laboratory for Systems Biology and Functional Genomics, Robert-Rössle-Str. 10, 13125 Berlin, Germany; 3 Institute of Chemistry and Biochemistry, Freie Universität Berlin, Laboratory of Protein Biochemistry, Thielallee 63, 14195 Berlin, Germany; 4 Center for Tumor Biology and Immunology (ZTI), Philipps-University Marburg, Hans-Meerwein-Straße 3, 35043 Marburg, Germany; 5 Leibniz-Institut für Molekulare Pharmakologie, Robert-Rössle-Strasse 10, 13125 Berlin, Germany; 6 Department of Biology, South University of Science and Technology of China, Shenzhen, Guangdong China

**Keywords:** Alternative splicing, 3′UTR, Protein stability, Protein disorder, Protein-protein interaction, Alternative open reading frame, Genome structure

## Abstract

**Background:**

3′-Untranslated regions (3′UTRs) play crucial roles in mRNA metabolism, such as by controlling mRNA stability, translation efficiency, and localization. Intriguingly, in some genes the 3′UTR is longer than their coding regions, pointing to additional, unknown functions. Here, we describe a protein-coding function of 3′UTRs upon frameshift-inducing alternative splicing in more than 10% of human and mouse protein-coding genes.

**Results:**

3′UTR-encoded amino acid sequences show an enrichment of PxxP motifs and lead to interactome rewiring. Furthermore, an elevated proline content increases protein disorder and reduces protein stability, thus allowing splicing-controlled regulation of protein half-life. This could also act as a surveillance mechanism for erroneous skipping of penultimate exons resulting in transcripts that escape nonsense mediated decay. The impact of frameshift-inducing alternative splicing on disease development is emphasized by a retinitis pigmentosa-causing mutation leading to translation of a 3′UTR-encoded, proline-rich, destabilized frameshift-protein with altered protein-protein interactions.

**Conclusions:**

We describe a widespread, evolutionarily conserved mechanism that enriches the mammalian proteome, controls protein expression and protein-protein interactions, and has important implications for the discovery of novel, potentially disease-relevant protein variants.

## Introduction

Recent work has highlighted the essential contribution of non-coding regions in controlling gene expression, especially in complex mammalian genomes [1]. In particular, 3′-untranslated regions (3′UTRs) play a crucial role in mRNA metabolism, e.g., by controlling mRNA stability, translation efficiency, and localization, or even as scaffolds to control protein localization [2–6]. Moreover, 3′UTRs emerge as essential regulatory elements in biological processes such as immune cell activation and tumorigenesis. In these settings, alternative cleavage and polyadenylation produce mRNA isoforms with shorter 3′UTRs that, due to the loss of microRNA-mediated repression, display increased protein expression [5, 6]. In tumor cells, elevated protein expression upon shortening of 3′UTRs is used to activate oncogenes or repress tumor-suppressor genes without mutating the genetic sequence [5, 7]. While these studies describe regulatory roles for 3′UTRs that do not affect the sequence of the expressed protein, some 3′UTRs are longer than their coding regions and could therefore fulfill additional, unknown functions [8]. Indeed, Fire and colleagues suggested that failure of the ribosome to terminate at stop codons can lead to translation into the 3′UTR. This resulted in a C-terminal extension of the investigated protein, which led to its destabilization; the authors suggest this to be a safety mechanism to quickly discard such aberrantly produced proteins [9]. Another report suggests that failure of ribosome recycling in yeast can result in re-initiation of translation after the canonical stop codon, leading to the expression of micropeptides [10, 11]. While these studies provide evidence for translation of short sequences from 3′UTRs, it remains unclear to what extent 3′UTRs can be expressed in mammals and if and how 3′UTR-encoded sequences are used in a regulated manner beyond a safety mechanism. Moreover, possible functionalities and a potential evolutionary conservation of 3′UTR-encoded amino acid sequences remain elusive.

Alternative splicing (AS) is a well-established mechanism that, through joining together different combinations of exons during mRNA maturation in over 90% of human multi-exon genes, multiplies the genome’s coding capacity and controls functionality at the molecular and the cellular level [12–14]. Deregulation of AS has been linked to various human diseases such as cancer and neurological disorders [15, 16], emphasizing its crucial regulatory function. So far, the analysis of AS has been almost exclusively directed towards frame-preserving splicing events, as frameshift-inducing AS is generally believed to induce nonsense-mediated mRNA decay (NMD) through generation of premature stop codons [17]. Thus, the coding information hidden in alternative reading frames and the potential regulatory function of isoforms encoded by these frames remain largely unexplored. In our work, we reasoned that transcripts resulting from frameshift-inducing AS of the penultimate exon escape NMD, as this leads to the usage of an alternative stop codon located in the last exon. Consistent with this idea, we have previously shown that frameshift-inducing AS of the penultimate U2af26 (U2AF1L4) exons 6 and 7 in mice allows regulated translation into the sequences supposedly representing the 3′UTR [18].

Here, we show that more than 10% of mouse and human genes contain splicing-accessible extended frames in their 3′UTR, and confirm translation in many cases using mass spectrometry of endogenous proteins. The resulting alternative C-termini control protein stability, likely through an elevated degree of protein disorder, and, in addition, show a strong enrichment for proline-rich protein-protein interaction motifs. Tissue- and development-specific AS of penultimate exons thus suggests dynamic control of protein levels and rewiring of interaction networks. Our data reveal that this mechanism is conserved across mammalian species, thus representing a general evolutionary strategy. Furthermore, as we demonstrate for a *retinitis pigmentosa*-causing mutation in the human phosphodiesterase PDE6G gene, misregulated translation into the 3′UTR is associated with the development of disease.

## Results

### Alternative splicing of penultimate exons frequently extends the ORF into the 3′UTR

**Fig. 1:**
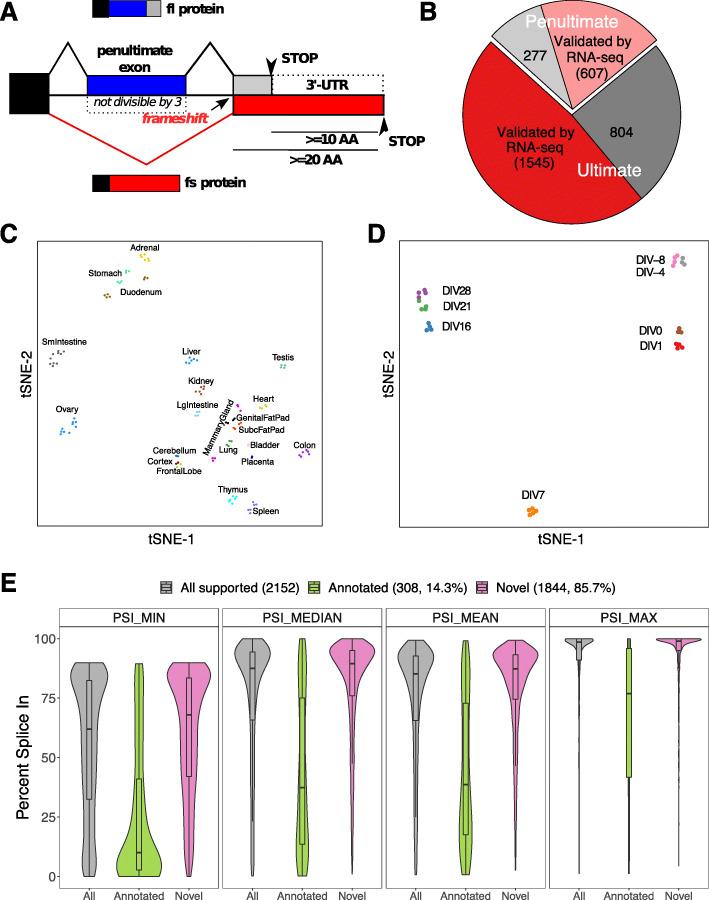
Alternative splicing of penultimate exons frequently extends the ORF into the 3′UTR. **a** Schematic representation of the identification of exon skipping events that allow translation into the 3′UTR. **b** Pie-chart showing validation of candidates by RNA-seq data for transcripts with stop codon in penultimate (light red: validated, light gray: not validated) or ultimate exon (red: validated, dark gray: not validated). **c** Two-dimensional tSNE plot showing tissue-specific penultimate exon skipping pattern using 22 mouse tissues. **d** tSNE plot showing neuronal differentiation stage specific penultimate exon skipping pattern. DIV, days in vitro. **e** Distribution of minimum (PSI_MIN), median (PSI_MEDIAN), mean (PSI_MEAN), and maximum (PSI_MAX) PSI values across different mouse tissues for penultimate exons that are alternatively spliced in at least one tissue (candidates from **b)** with RNA-Seq support), either for all exons or divided into annotated and novel isoforms

In protein coding genes, stop codons are located either in the last or in the penultimate exon, as they will, in many cases, trigger NMD if present in internal exons or in the penultimate exons more than 50 nucleotides upstream of the exon-exon junction. Regulated alternative splicing, for example intron retention, AS within the 3′UTR or frameshift-inducing skipping of internal exons leading to the generation of a stop codon in the following exon, is a widely used mechanism to control gene expression [19]. However, skipping of a penultimate exon either containing the stop codon, or bringing the canonical stop codon in the last exon out of frame, will not induce NMD, as the new stop codon will be/remain in the last exon. This could provide the means to extend the translatable sequence of an mRNA into the sequence that supposedly represents the 3′ UTR. To establish whether splicing-induced translation into the 3′UTR is a general mechanism, we analyzed skipping of the penultimate exon and its effect on the reading-frame in the mouse transcriptome (Fig. 1a). We followed the pipeline in Additional file 1: Fig. S1A to identify genes where skipping of the penultimate exon induces a frameshift that results in at least 20 new amino acids (AA) with ≥ 10 AA encoded in the original annotated 3′UTR. Three thousand two hundred thirty-three transcripts from 2860 gene loci were identified, representing > 10% of all protein-coding genes. Importantly, of the 3233 penultimate exons, we found 2152 (66.6%) to be skipped (percent spliced in, PSI < 90) in at least one mouse tissue, based on publicly available RNA-seq data (Fig. 1b, Additional file 2: Table S1), and the majority of these AS events (85.7%, 1844/2152) was not previously annotated (Additional file 2: Table S1). Interestingly, skipping of penultimate exons is controlled in a highly tissue-specific manner, as observed by comparing their splicing pattern across 22 mouse tissues (Fig. 1c, Additional file 1: Fig. S1B, C). In addition, AS of penultimate exons is, as observed for other AS events, a dynamic process, as their splicing pattern changes during neuronal differentiation (Fig. 1d, Additional file 1: Fig. S1D, E, [20]). These data suggest that frameshift-inducing skipping of penultimate exons is used as part of the regular splicing program, which is further supported by the high detection rate of penultimate exon skipping in RNA-Seq data (Fig. 1e, Additional file 1: Fig. S1F). The detection rate of penultimate exon skipping was significantly higher than for internal frame-preserving or frameshifting skipping events (*p* < 2.2 × 10^−16^), and this held true for different cutoffs for an exon to be considered alternative (Additional file 1: Fig. S1F). This trend was also true for skipping of frame-preserving penultimate exons and skipping of exons that would lead to a shorter C-terminus, suggesting that penultimate exons are in general more alternatively spliced (Additional file 1: Fig. S1F). For the following analyses, we have focused on the cases where skipping extends the reading frame by at least 20 amino acids with 10 of them in the sequence supposedly representing the 3′UTR (Fig. 1a). Analyzing the meta-exon PSI distribution suggests that for the majority of novel (not previously annotated) penultimate exon skipping events, the respective exons are part of the canonical mRNA and only skipped in selected tissues. In contrast, the previously annotated cases have lower PSIs, which could indicate that the skipping isoform is the major variant, whereas inclusion shortens the protein (Fig. 1e). To gain first insight into a possible concerted regulation of frameshift-inducing alternative splicing events, we performed a motif analysis of exons strongly regulated during neuronal differentiation (ΔPSI > 50 between any two time points). This revealed an enrichment of binding motifs for Pcbp3 and Mbnl1, two RBPs that are strongly upregulated during neuronal differentiation (Additional file 1: Fig. S1G, H, [20]). Together, these analyses establish extension of the reading frame past the canonical stop codon via frameshift-inducing AS as a dynamic and widespread regulatory mechanism that expands the translatable sequence into parts of the genome that were previously considered to be non-coding.

**Fig. 2:**
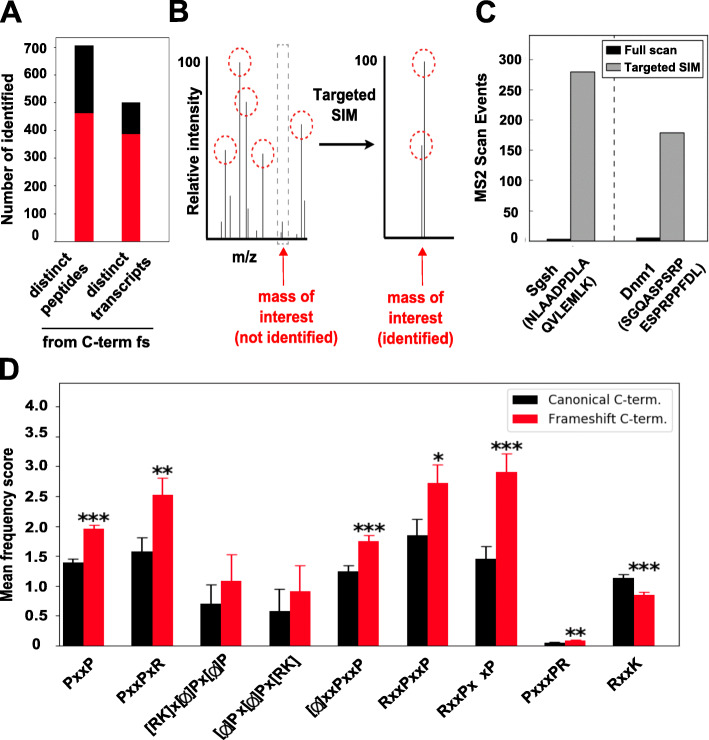
Frameshift-isoforms are translated and are enriched in PxxP motifs. **a** Number of unique peptides identified in mouse brain lysate mapping to predicted frameshift C-termini (unambiguously identified frameshift C-termini marked in red); some peptides map to the same frameshift sequences. **b** Schematic representation of targeted SIM MS method. Peptide mass peaks with the highest relative intensities (red circles) are chosen for MS2 fragmentation and subsequent identification. By focusing on only a narrow mass range, specific peptide mass peaks can be targeted and, due to decreased background, fragmented and further identified. **c** Targeting of peptide NLAADPDLAQVLEMLK, a proteotypic peptide mapping to the novel frameshift C-terminus of SGSH, and SGQASPSRPESPRPPFDL, a peptide mapping to the predicted frameshift isoform of DNM1, which has been annotated as isoform 4, using targeted SIM MS. **d** Frequency of SH3-domain-binding proline-rich motifs in mouse full-length C-termini (black) and frameshift C-termini fulfilling the criteria defined in Fig. 1a (red)

To confirm expression of the 3′UTR-encoded variants on the protein level, we performed mass spectrometric analysis of mouse brain lysate. To identify peptides mapping to the C-terminus of frameshift-isoforms, we generated a custom database by supplementing the mouse Uniprot database with frameshift-isoforms containing expected C-terminal sequences. Of the 7887 theoretical tryptic peptides mapping to frameshift C-termini, we identified 705 distinct peptides mapping to frameshift C-termini from 482 distinct transcripts (Fig. 2a, Additional file 3: Table S2). Four hundred sixty-one of these peptides are proteotypic, i.e., unambiguously identifying 380 frameshift-isoforms. A targeted MS approach (SIM, see Experimental procedures for details, Fig. 2b, c) dramatically increased the identification of proteotypic peptides, suggesting that many additional frameshift-peptides are masked by mass peaks of more abundant species of a similar mass in the proteome-wide analysis. We thus consider the number of identified 3′UTR-encoded C-termini in Fig. 2a to be strongly underestimated. However, this analysis clearly demonstrates that the mechanism we describe here, frameshift-inducing skipping of penultimate exons resulting in translation into supposed “3′UTRs,” is used in vivo, thus representing a new and unexpected example how non-coding parts of the genome can become coding, thereby further increasing the genome’s coding capacity.

### Enrichment of PxxP motifs and disorder-promoting proline residues in 3′UTR-encoded sequences

To globally investigate the functionality of the new C-termini, we analyzed the frequency of known functional AA motifs. Interestingly, we observe a specific and significant enrichment of different proline-rich PxxP motifs in the frameshift C-termini (Fig. 2d, Additional file 3: Table S2). PxxP motifs represent well-known binding sites of SH3-domain-containing proteins and form interaction hubs that are essential for a variety of signaling pathways crucial for cellular functionality, such as the Ras or PI3-kinase signaling networks. Further, the nature of specific proline-rich sequences (e.g., class I, [RK]x[phi]Px[Phi] P, and class II, [phi]Px[phi]Px[RK]; see Fig. 2d) can have variable effects on interaction strength and specificity, allowing an even finer-grained degree of control [21]. Thus, introduction of PxxP motifs into frameshift proteins in a splicing-dependent and tissue-specific manner creates novel opportunities to rewire signaling networks, which is in line with the recent finding that splicing isoforms of the same protein often have dramatically altered interactomes [22].

**Fig. 3:**
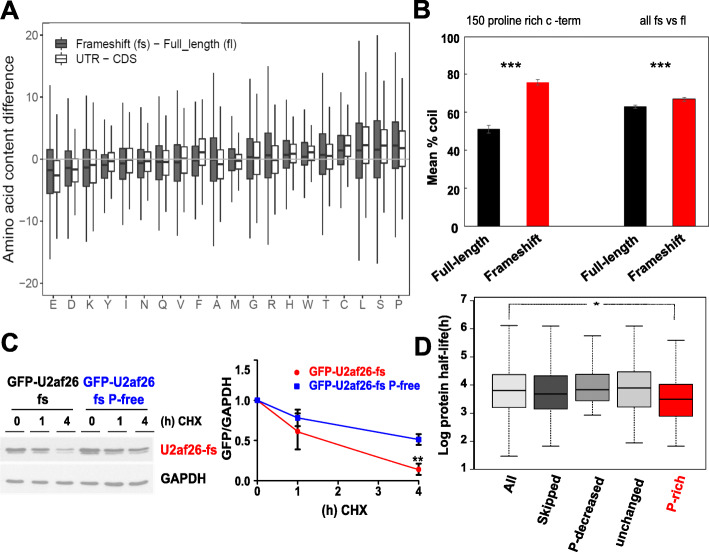
Translated frameshift-isoforms display an increased proline content resulting in reduced protein stability. **a** Boxplot comparing the AA content between frameshift and full-length sequences (gray boxes) and between the sequences 100 AA before (CDS) and after canonical stop codons (UTR, white boxes, see Experimental procedures for details). **b** Proportion of predicted coiled (unstructured) secondary structural elements in 150-full-length (black) and frameshift (red) mouse C-termini with the largest increase in proline content from full-length to frameshift (left) or all fl and fs C-termini (right). Only fs isoforms confirmed by RNA-seq are included. **c** Protein stability of GFP-tagged fs U2AF26 or a proline-free variant (in U2AF26 the fs frame is accessible through skipping of two neighboring exons, 6 and 7, exon 7 being the penultimate one). Translation was inhibited by cycloheximide (CHX) and protein degradation was determined by immunoblotting, *n* = 3. **d** Protein half-life in 3T3 cells of all measured proteins (All, *n* = 3573) and genes from the frameshift list (Additional file 2: Table S1) with PSI < 90 (Skipped, *n* = 192). Spliced genes were further divided in three groups based on the proline content of the alternative frame: (i) lower compared to the original frame (*P*_fl_-*P*_fs_ > 3, P-decreased *n* = 36), (ii) similar proline content (3 > *P*_fl_-*P*_fs_ > − 3, P-unchanged, *n* = 76), and (iii) higher proline content (*P*_fl_-*P*_fs_ < − 3, P-rich, *n* = 80) (*p < 0.05)

The generation of new PxxP motifs in the alternative C-termini prompted us to compare the overall AA content between full-length (fl) and frameshift (fs) isoforms. The new protein sequences showed strongly elevated cysteine, serine, leucine, and proline content when compared to the canonical C-termini (Fig. 3a, gray bars); codons for these amino acids were generally enriched in 3′UTR sequences (Fig. 3a, white bars, also see [9]). Proline showed the strongest enrichment in the frameshift C-termini, and a similar enrichment of proline was found in dual coding regions, where an elevated proline content positively correlated with intrinsic protein disorder [23]. Consistently, a prediction of the secondary structural elements of the frameshift and full-length C-terminal sequences with increased proline content revealed a significant increase in coiled structures in 3′UTR-encoded frameshift C-termini (Fig. 3b). As peptide sequences in a random coil state sample all possible degrees of freedom in their conformational space, they are thermodynamically considered to represent protein disorder [24]. We thus observe a dramatic increase in protein disorder among the frameshift C-termini with an increased proline content. When comparing all full-length and frameshift sequences, we still observe a significantly increased disorder in fs C-termini, but the difference was smaller than for the frameshift proteins with high proline content (Fig. 3b). Since proteins with intrinsically disordered regions have been suggested to possess shorter half-lives, likely due to rapid proteasomal degradation [25], we hypothesized that an increase of the degree of disorder in the 3′UTR-encoded C-termini, possibly through increased proline content, could lead to reduced protein half-life as a general feature of frameshift-isoforms.

### 3′UTR-encoded C-terminal extensions reduce protein stability and abundance

To investigate a potential shared functionality conferred by the increase in proline residues in the frameshift isoforms, we compared the expression of several frameshift C-termini to the respective canonical proteins. In all cases tested, the 3′UTR-encoded C-terminal extensions resulted in proteasome-dependent destabilization and strongly reduced basal expression of the protein (Additional file 1: Fig. S2A-D, see below for further examples), whereas the mRNA expression was not decreased (Additional file 1: Fig. S2E). This finding is in line with data showing that 3′UTR-encoded sequences, generated due to translational readthrough, destabilize the resulting proteins [9]. Consistent with a key role of the high proline content in destabilization, a proline-free variant of the alternative U2af26 C-terminus is significantly stabilized (Fig. 3c). The reduced basal expression of the alternative C-termini may additionally be affected by the use of suboptimal codons [26, 27], as codon optimization increased the expression level without altering protein stability (Additional file 1: Fig. S3A, B). To provide further evidence for a global correlation of higher proline content and reduced protein stability, we performed a system-wide analysis in mouse 3T3 cells. Using publicly available RNA-seq data [28], we identified 603 penultimate exons in 564 gene loci from our list of frameshift-inducing splicing events (Additional file 4: Table S3) that showed a PSI value of < 90. We then analyzed the stability of the corresponding proteins based on previously published protein half-life data [29]. Of the 564 gene loci with a PSI value < 90, protein half-life was determined for 192 proteins. While the half-life of the 192 alternatively spliced candidates was not generally reduced when compared to all proteins measured, those with an increased proline content in the alternative C-termini demonstrated significantly lower stability (Fig. 3d, Additional file 4: Table S3). This effect is likely underestimated, as we cannot differentiate between the frameshift and the canonical protein isoforms in our half-life analysis. Together, these data establish an elevated proline content, increased protein disorder, and reduced stability as common features of the alternative 3′UTR-encoded C-termini. This mechanism could act as surveillance pathway to substitute for NMD, as an inherent limitation of NMD is that it does not recognize erroneous skipping of frameshift-inducing penultimate exons and the cell will have to deal with the resulting products at the protein level. On the other hand, not all extended C-termini will reduce protein stability, and therefore, regulated skipping of frameshift-inducing penultimate exons will have additional functions, e.g., in rewiring protein-protein interaction networks as discussed above (and see below).

### AS-induced translation into the 3′UTR, leading to an increase in proline content and PxxP motifs, is conserved across mammals

**Fig. 4:**
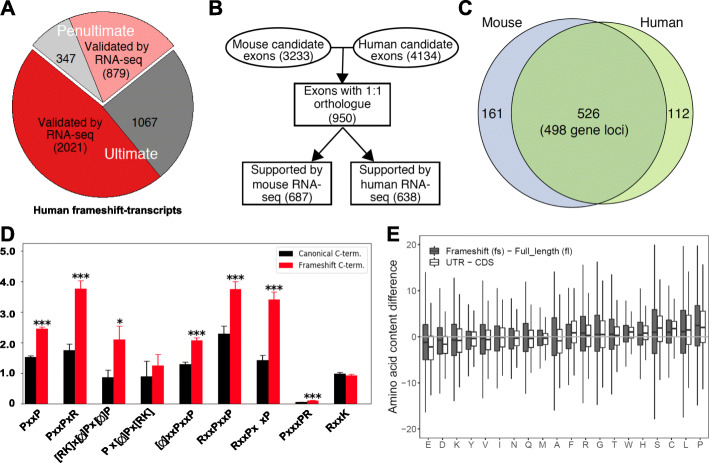
Conservation of proline-rich alternative frames encoding PxxP motifs. **a** Identification and validation of human frameshift candidates in RNA-seq data. **b** Schematic illustration of the identification of conserved frameshift-inducing penultimate exons between human and mouse. **c** Venn diagram showing a significant overlap (hypergeometric test, *p* < 1.3 × 10^−22^). **d** Frequency of SH3-domain-binding proline-rich motifs in human full-length C-termini (black) and frameshift isoforms fulfilling the criteria defined in Fig. 1a (red). **e** Boxplot comparing the AA content differences between frameshift and full-length frames (gray boxes), and between the sequences 100 AA before (CDS) and after canonical stop codons (UTR, white boxes)

To investigate cross-species conservation of frameshift-induced translation into the 3′UTR, we applied our pipeline (Additional file 1: Fig. S1A) to the human transcriptome. Four thousand three hundred fourteen candidates where skipping of the penultimate exon extends the reading frame into the 3′UTR were identified, with 2900 showing evidence for AS in RNA-seq data (Fig. 4a, Additional file 2: Table S1). These AS events are again highly regulated, as many candidates show a tissue-specific expression pattern (Additional file 1: Fig. S4A, B). Moreover, frameshift-inducing skipping of the penultimate exon is conserved across mammals, as we find a substantial overlap between mouse and human skipping events in cases where we can identify a 1:1 orthologue (Fig. 4b, c, Additional file 2: Table S1). These conserved genes show significant enrichment in modulation of chemical synaptic transmission or glutamate receptor signaling pathway and others (Additional file 1: Fig. S4C), pointing to an evolutionarily conserved function in mammalian development. We do however find that frame preserving penultimate exons and the exons where skipping leads to shorter C-termini show higher evolutionary conservation, especially at the level of 1:1 orthologues (Additional file 1: Fig. S4D). A possible explanation is that the longer, splicing-accessible C-termini have evolved independently and thus could contribute to species-specific traits.

**Fig. 5:**
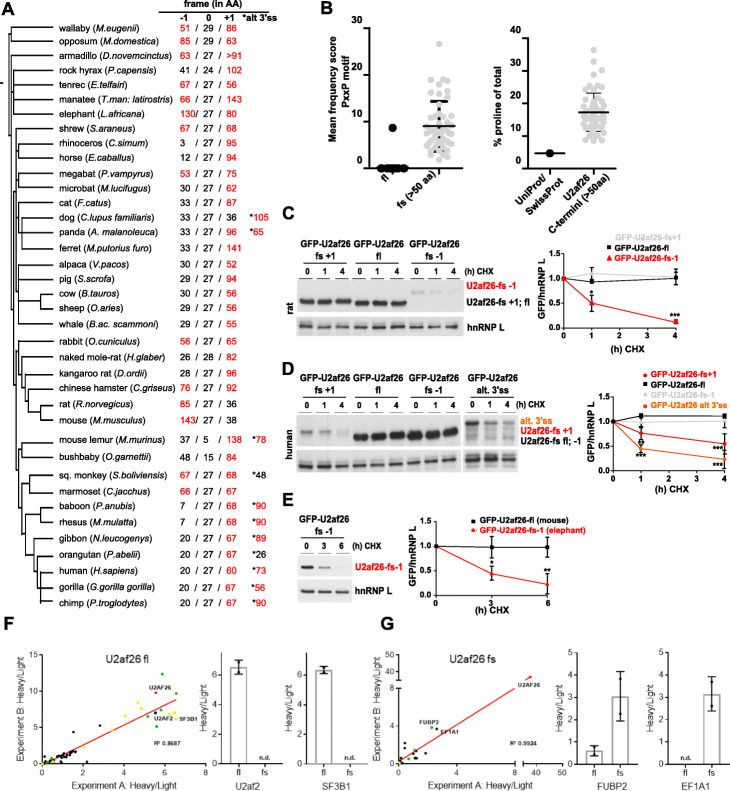
Evolutionary conservation of unstable, proline-rich U2af26 frames encoding PxxP motifs. **a** Evolutionary tree of mammalian species harboring a U2af26 gene generated with PhyloT. For each species the length of possible ORFs in all three hypothetical frames encoded by the last U2af26 exon are depicted; the annotated C-terminus of U2af26 is defined as frame 0. Potential ORFs with more than 50 AAs are highlighted in red. The asterisks mark frames accessible via an alternative 3′ss. **b** Frequency of SH3-domain-binding proline-rich PxxP motifs in extended − 1 or + 1 frames of U2af26 in comparison to the fl frame 0 (left panel). Proline content of extended U2af26 alternative frames compared to the UniProt/SwissProt average representing all proteins (right panel). **c**–**e** Stability of N-terminally GFP-tagged U2af26 frames from different species: protein stability of all possible rat (**c**) and human frames (**d**), including the frame accessible through an alternative 3′ss, and the alternative extended elephant frame − 1 (**e**). Stability was determined as in Fig. 3c (*n* > 3). (*p* < 0.001: ***, *p* < 0.01: **, *p* < 0.05: *). **f**, **g** Enrichment of interaction partners of full-length (**f**) and frameshift (**g**) isoforms of U2af26-GFP by quantitative mass spectrometry after GFP pulldown. Interaction partners are enriched with heavy/light (bait/bead control) intensity ratios, two independent experiments are plotted to show reproducibility. GFP-tagged U2af26 is shown in red; proteins known to be involved in splicing are shown in green and previously identified proteins that interact with or are part of the U2snRNP are shown in yellow. Two examples for specific interaction partners are shown

The human frames show an enrichment in PxxP motifs that is very similar to the pattern observed in mouse, and as observed in mouse, we find a specific increase in overall proline content (Fig. 4d, e). As for the analysis in mouse, this correlates with an increase in disorder of the frameshift C-termini, especially in the ones with increased proline content (Additional file 1: Fig. S4E). These findings provide strong evidence for a functional conservation and suggest that these features of the alternative frames are beneficial during evolution as they are conserved across species. To further extrapolate our findings to other mammals, we used U2af26 as a model gene and investigated the presence of alternative 3′UTR-encoded reading frames. Remarkably, in all mammalian species that harbor a U2af26 gene, the last exon contains, in addition to the canonical frame, at least one extended, 3′UTR-encoded frame (≥ 50 AA) that is accessible through a − 1 and/or + 1 frameshift (Fig. 5a, Additional file 5: Table S4). In addition to skipping of the penultimate exon, an alternative 3′ splice site (3′ss) emerged as a further mechanism to access an alternative reading-frame in the supposed 3′UTR (therefore escaping the pipeline described in Additional file 1: Fig. S1A). This alternative 3′ss is highly conserved in primates (Additional file 1: Fig. S5A), and its usage was previously demonstrated for human U2af26 [18].

While the extended U2af26 frames display no overall sequence conservation (Additional file 1: Fig. S5B and data not shown), they do share common features. While PxxP motifs are absent in nearly all fl U2af26 proteins, the extended fs C-termini of all species encode at least one, and in most cases many, PxxP motif (Fig. 5b, left). In addition, elongated U2af26 C-termini from all species show a strong enrichment of proline when compared to the average of the UniProt/SwissProt database (Fig. 5b, right, Additional file 1: Fig. S5C). Consistent with a destabilizing function of an elevated proline content, we observed a severely reduced protein stability of extended rat, human, and elephant U2af26 alternative frames (Fig. 5c–e). In contrast, canonical frames and hypothetical short alternative frames (< 50 AA) are stable (Fig. 5c, d; for the respective mouse frames, see Additional file 1: Fig. S2A and Fig. S5D). As for all alternative C-termini, the destabilization of extended rat, human, and elephant frames was dependent on the proteasome (Additional file 1: Fig. S6A-D). In addition, we investigated the effect of the frameshift C-terminus on the mouse U2af26 interactome using mass spectrometry. Consistent with its function in 3′ splice-site recognition, the U2AF26 full-length protein mainly interacts with splicing factors including its dimerization partner U2af2 and U2snRNP-associated proteins (Fig. 5f, Additional file 6: Table S5). In contrast, for the U2af26 frameshift protein, interactions with these splicing factors are completely abolished (Fig. 5g). Loss of the interactions of the U2af26 frameshift protein with splicing factors is consistent with its cytoplasmatic localization and splicing-independent function [18]. Instead, novel interactions were identified, demonstrating that the splicing-induced frameshift almost completely rewires the interactome. Consistent with the generation of 3′UTR-encoded PxxP motifs, we find an enrichment of identified (but, due to low abundance, not quantified) peptides originating from SH3 domain-containing proteins in the IP with the frameshift protein (Additional file 6: Table S5, marked in yellow). Altogether, these data reveal AS-induced translation into the 3′UTR as a means to control protein stability and protein-protein interaction motifs as a general mechanism that is conserved across mammalian species.

### A *retinitis pigmentosa*-causing mutation in PDE6G leads to frameshift-induced translation of the 3′UTR

**Fig. 6:**
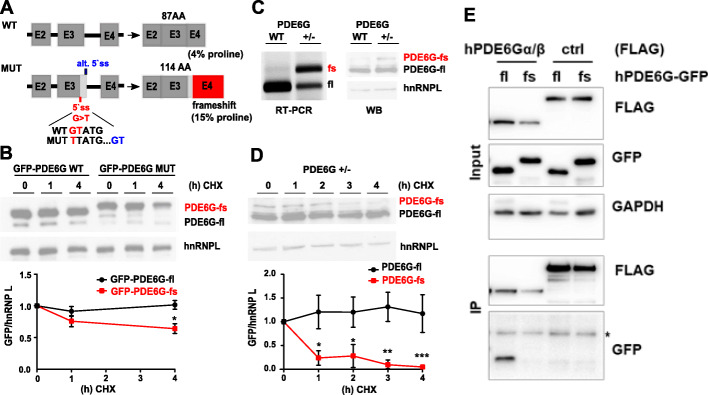
A *retinitis pigmentosa*-causing mutation in the human PDE6G gene causes a frameshift that adds a proline-rich, destabilizing C-terminus with altered protein-protein interactions. **a** Schematic representation of the wildtype (WT) and mutated (MUT) PDE6G gene. Alternative ss (blue) usage causes a frameshift and translation into the 3′UTR. Strategy for CRISPR/Cas9-mediated insertion of the mutation is shown (see Experimental procedures for details). **b** Stability of GFP-tagged fl and fs PDE6G C-termini was analyzed as in Fig. 3c. *n* = 3. **c** RT-PCR and immunoblotting of either WT Hek293T cells or cells heterozygous for the PDE6G mutation shown in a. **d** PDE6G +/− cells were treated as indicated and stability of PDE6G isoforms was determined as in Fig. 3c using a PDE6G specific antibody, *n* = 3. **e** Specific co-precipitation of PDE6α/β-Flag with GFP-PDE6G-fl but not GFP-PDE6G-fs. The N-terminus of the unrelated RNA helicase Brr2 served as Flag-tagged control protein. * marks IgG

Finally, an extended analysis of human PDE6G, a phosphodiesterase subunit involved in phototransduction [30], underlines the clinical relevance of frameshift-proteins. The human PDE6G gene harbors an extended 3′UTR-encoded frame accessible through a cryptic 5′ss in the penultimate exon. Based on minigene analysis, a *retinitis pigmentosa*-causing mutation in the canonical 5′ss was suggested to result in usage of this frameshift-inducing alternative 5′ss (Fig. 6a and [31]), but the molecular mechanism of the disease development remains unclear. Consistent with our previous findings, the 3′UTR-encoded C-terminus of the mutant protein displays a strongly elevated proline content (Fig. 6a) and reduced protein stability when fused to GFP (Fig. 6b). To assess endogenous protein stability, we inserted the disease-causing PDE6G mutation in Hek293T cells using CRISPR-Cas9-mediated genome editing resulting in a heterozygous cell line with one wt and a mutated allele (Fig. 6c). This confirmed the usage of the cryptic 5′ss and translation of an extended frameshift-protein in the presence of the mutation at the endogenous level (Fig. 6c). Consistent with our model, in a heterozygous clone the stability of the frameshift-protein was strongly reduced (Fig. 6d). Intriguingly, in CoIPs, the mutant protein also demonstrates lost binding to the known interaction partners PDE6A/B (Fig. 6e). Hence, similar as for U2AF26 (Fig. 5f, g), the human PDE6G frameshift protein further confirms frameshift-induced alteration of protein-protein interactions (Fig. 6e). Since the interaction of PDE6G with PDE6A/B is crucial for the integrity of the phosphodiesterase holoenzyme [32], dysregulated complex formation, as observed for the PDE6G frameshift variant, is expected to contribute to the development of the disease phenotype [30, 33, 34].

## Discussion

**Fig. 7:**
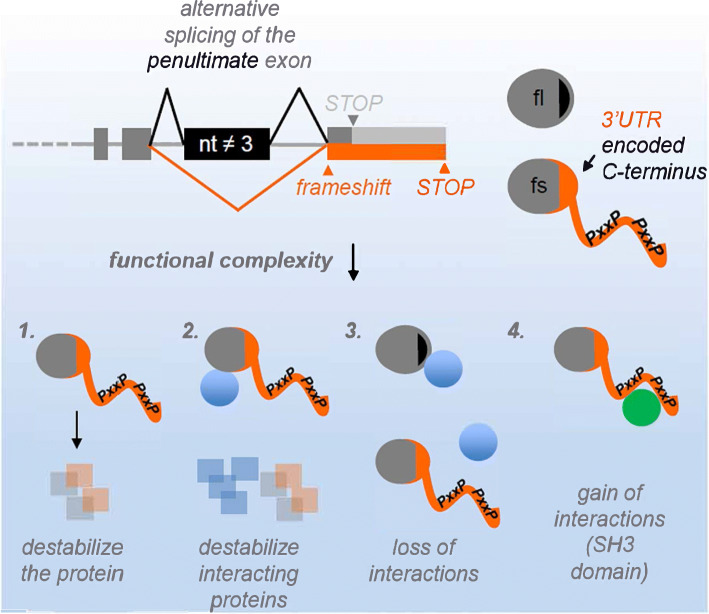
Frameshift-inducing alternative splicing generates protein isoforms with diverse functionalities. Alternative splicing of penultimate exons frequently extends the upon reading frame into the supposed 3′UTR. The resulting protein variants are identical to the full-length versions except a novel C-terminus. The novel C-termini are enriched in proline and PxxP motifs (top). This can (1) destabilize the resulting protein, (2) destabilize the protein together with interaction partners, and (3+4) modify protein-protein interactions

Alternative splicing is a well-established mechanism that multiplies the genome’s coding capacity, as, for example, over 90% of human multi-exon genes express various isoforms, often in a tissue-specific manner [12, 13, 35]. However, the potential of AS to extend the translated sequence past the canonical stop codon into the 3′UTR has so far been overlooked. Here we report the existence of a large number of mammalian AS-induced frameshift events, resulting in the expression of distinct, C-terminally extended isoforms. We find two features of these extended C-terminal sequences: (a) they show a significantly higher frequency of protein-protein interaction motifs, which may contribute to rewiring of protein-protein interaction networks, and (b) in many cases they act in a destabilizing manner (Fig. 7). We suggest that the latter is due primarily to elevated content of proline residues leading to higher levels of protein disorder. As the respective mRNAs are not recognized by NMD, this could represent a surveillance pathway to correct splicing errors, but it could also contribute to control protein levels in a regulated, e.g., tissue-specific, manner.

Although we have already found more than 10% of mouse and human protein-coding genes to contain splicing-accessible, translatable 3′UTRs, we expect this percentage to be underestimated. In several species, we find an alternative 3′ss in the last U2af26 exon to induce a frameshift and translation into the 3′UTR and in human PDE6G an alternative 5′ss in the penultimate exon has the same effect. Moreover, skipping of the second and third but last exons (again shown for U2af26) or other internal exons may also lead to a frameshift without inducing NMD, thus further increasing the number of genes with translated 3′UTRs. Given these additional mechanisms, we expect that the percentage of genes in which the “3′UTR” is used as coding sequence is very high, probably representing the norm rather than an exception. Consistently, recent work has highlighted the presence of translatable sequences in parts of the genome that were considered non-coding [36, 37].

Proline-rich sequences are the most common protein interaction motifs in higher eukaryotes [38], and this is paralleled by a strong expansion of SH3 domains in eukaryotic signaling proteins [39]. The most frequent SH3-binding motifs, RxxPxxP and PxxPxR, are both overrepresented in the frameshift sequences in mouse and human, pointing to an evolutionary selection mechanism generating novel SH3-domain-based interactions in 3′UTR-encoded sequences. Multivalency of proline-rich sequences is a principle known to enhance protein-complex formation, either strengthening a particular interaction [40], or allowing proteins to cluster into larger, yet still dynamic, assemblies [41]. Thus, introduction of PxxP motifs into frameshift proteins creates novel opportunities to rewire signaling events, e.g., by affecting Ras or PI3-kinase signaling pathways. This is in line with the recent finding that splicing isoforms of the same protein often have dramatically altered interactomes [22] and consistent with our data showing altered interaction networks of fl and fs U2AF26. Interestingly, expression of isoforms from dual coding regions within the ORF was previously described to result in the loss of critical functional domains [23]. In contrast, splicing-regulated 3′UTR translation that generates additional functional AA motifs could introduce new functions through the formation of novel protein-interaction domains. In addition to the generation of new protein-protein-interaction motifs, our analysis identifies a high proline content as a common feature of the 3′UTR-encoded C-termini that results in reduced stability of the frameshift proteins. Moreover, an elevated proline content correlates with increased proportions of coiled, unstructured regions in the alternative C-termini, which is consistent with earlier findings that disordered regions facilitate proteasomal degradation [25]. Hence, we propose that an enrichment of disorder due to an elevated proline content in the 3′UTR-encoded C-termini reduces the protein half-life of the frameshift isoforms.

We suggest a model in which 3′UTR-encoded frameshift isoforms, despite their degradation in a proteasome-dependent manner, display functionality in a gene-context-dependent and tissue-specific manner. Whereas in some genes, frameshift-inducing AS may simply result in quick degradation of unproductive variants, as described for translational readthrough [9], we expect that in many cases the 3′UTR-encoded sequences are functionally important. This idea is also supported by the considerable length of the alternative C-termini, which in many cases add more than 100 amino acids, suggesting a function beyond a simple degradation signal. Furthermore, in frameshift proteins, the original sequence, including functional domains, is preserved up to the penultimate exon. Thus, expression of a destabilizing 3′UTR-encoded C-terminus, regulated by the degree of frameshift-inducing exon skipping, may control the overall half-life of the protein and adjust it according to cellular demands in a dynamic or tissue-specific manner. An intriguing example for the regulatory role of a frameshift protein that goes beyond destabilization is the U2af26 gene. In contrast to the stable, nuclear, full-length U2af26, the frameshift-isoform is an unstable, cytoplasmic protein generated by circadian-like exon skipping, thus controlling gene expression in space and time. Importantly, the frameshift U2af26 protein co-destabilizes its interaction partner Per1, allowing control of the core machinery of the circadian clock [18]. We find this destabilization to be conserved across species (Additional file 1: Fig. S7), emphasizing its functional role throughout evolution.

Frameshift-induced translation into the 3′UTR may prove generally important in the etiology of genetic diseases. We find that a *retinitis-pigmentosa* causing mutation in human PDE6G leads to the expression of a 3′UTR-encoded frameshift-isoform that is both destabilized and shows an inhibited ability to interact with its known binding partners PDE6A and PDE6B. Since the function and protein-protein interactions of the PDE6G subunit are crucial for the integrity of the phosphodiesterase holoenzyme, expression of a mutant PDE6G frameshift protein is expected to contribute to the disease phenotype. We expect that the mutant PDE6G protein represents only the first example of so far unexplored, pathogenic C-terminally extended isoforms, linking the dysregulation of frameshift events with disease.

Together, our observations lead us to propose AS-induced frameshifts and translation into the 3′UTR as a general evolutionary strategy. Generating frameshift isoforms in this way provides an energetically inexpensive mechanism for evolution to experiment with novel functions and protein-protein interactions. Furthermore, the reduced stability of frameshift C-termini may allow a degree of protection against disadvantageous binding events, thus selecting for frameshift-variants generating stabilizing interactions. It also allows the precise control of protein half-life in a tissue-specific or time-of-day-dependent manner, as shown for U2af26. From an evolutionary perspective, the use of the 3′UTR as a coding sequence is well suited, as there is no pressure to maintain a coding frame, and much of the original functionality of the protein is presumably preserved. These observations suggest a unique mechanism by which AS further expands and enriches the diversity of the mammalian proteome, thus giving the evolutionary process the opportunity to exploit and experiment with novel protein functionalities.

## Experimental procedures

### Bioinformatics analysis

#### Penultimate exon annotation

Reference sequences and gene annotations of the mouse and human genome were downloaded from the Ensembl FTP server (ftp://ftp.ensembl.org/pub/release-98/). Only protein-coding transcripts were used in the analysis. To check whether skipping of the penultimate exon leads to a different open reading frame, transcripts (1) with an annotated stop codon in the last exon and a penultimate exon length not divisible by three (“Ultimate”); or (2) with annotated stop codon in the penultimate exon (“Penultimate”) were considered. To rule out double detections, penultimate exons in different transcript isoforms, but with the same (1) end position of upstream exon, (2) start position, (3) end position, (4) start position of downstream exon, and (5) stop codon position, were counted only once. Given the genomic sequences and gene annotations, transcriptome sequences of annotated frames and new frames after skipping were built using custom Perl scripts. The translation of nucleotide sequences into AA sequences was performed using custom Perl scripts. Only the transcripts with at least 20 AAs gained and at least 10 AAs extended beyond the annotated stop codon after exon skipping were retained for further RNA-seq analysis

### RNA-seq analysis

We used publicly available RNA-seq data for our analyses (see the Availability of the data and materials for details). These data were aligned to the corresponding reference sequences using TopHat2 with default parameters and Ensembl gene annotation (v2.1.1). The candidate penultimate exons from *Penultimate exon annotation* section were converted to GFF format. Mixture of Isoforms (MISO) Bayesian Inference model (v0.5.4) was then used to infer the splicing pattern of these candidates across different samples.

For each sample, exons with ≥ 20 supporting reads are considered to be expressed and retained for further analysis. For each candidate exon in a given tissue, the average PSI (percent spliced in) value of all expressed samples in this tissue is used. Alternative splicing of a candidate exon is considered to be supported by RNA-seq if it is expressed and alternatively spliced (PSI < 90) in at least one tissues. ComBat in sva package (v3.31.1) was used to correct the batch effect for 22 mouse tissues which were sequenced using different library protocols. For unsupervised t-SNE analysis, pairwise sample distance was calculated by using 1 minus weighted Pearson correlation coefficient as the distance measure. We used median absolute deviation of the PSI values as the weight, giving more variable exons greater influence on tissue specificity. The resulting distance was used to perform the *t*-SNE analysis (Rtsne package v0.15).

For comparison, we also analyzed internal frame-preserving (Internal_fp), internal frameshifting (Internal_fs), and penultimate exon that are either frame-preserving (Penulti_fp), or frame shifting leading to a shorter alternative C-terminus (Penulti_short) using the same strategy.

### RBP motif analysis

We used the neuron differentiation dataset and retained 1635 exons that are expressed in all differentiation stages to exclude gene expression effect. We selected as BACKGROND 895 exons that are constitutively spliced in (PSI > 90) or constitutively spliced out (PSI < 20) in every sample. We also selected 72 exons with ΔPSI > 50 between any two stages for RBP binding motif enrichment analysis. To do this, we used the sequences of these penultimate exons as well as their 50 bp upstream and downstream introns as input for Homer with the following command:

*findMotifs.pl TARGET.fa fasta OUTPUT -fastaBg BACKGROUND.fa -rna -len 6,7,8*

Then we retained the motifs with (1) *p* value < 1e−5, (2) frequency in TARGET > 15%, and frequency in BACKGROUND < 5% to match the motifs annotated in the RBPmap database (http://rbpmap.technion.ac.il/index.html).

### Human-mouse orthologue exons

The coordinates for each penultimate exon were marked using two lines in BED format, for upstream and downstream introns, respectively. The coordinates for human (mouse) were then converted to the corresponding locations in the mouse (human) genome using the UCSC liftOver tool. Only the penultimate exons with 1:1 orthologue relationships [42] as well as at least 20 AAs gained and at least 10 AAs extended were retained for conservation analysis. Gene ontology analysis for the conserved exons between human and mouse was performed using https://amp.pharm.mssm.edu/Enrichr/.

### Protein half-life

The half-life for each protein in mouse 3T3 cells was downloaded from our previous study [29]. Only the genes with measured protein half-life were retained for further analysis. The gene numbers for each group are 3573, 192, 36, 76, and 80, respectively (see main text for group classification). Only the genes with skipped penultimate exons, extended AA, and increased proline content showed significantly shorter half-life compared to all the expressed genes.

### AA composition

The AA content in the original frame was calculated as the number of a certain AA divided by the total number of AAs coded by the last two exons of the transcript. The AA content in novel frame was calculated as the number of a certain AA divided by the total number of AAs encoded by the new frame of the last exon. To calculate the AA content in the annotated coding region, the last 100 AAs of the annotated frame were considered. To calculate the content in annotated 3′UTR, the first 100 AAs of all three possible frames after the stop codon were used even if new stop codons occurred, and the average content of the three frames was calculated.

### Cross-species conservation of the U2af26 gene

The last exon of the U2af26 gene of different mammalian species was identified using sequences available at the UCSC Genome Browser website. If not annotated, the last exon was identified by alignment of the genomic sequence to the last exon of a closely related species. The last exon was translated in all three potential forward frames, and the canonical C-terminus of U2af26 was defined as frame 0 (see Additional file 5: Table S4 for sequences). Extended alternative frames of at least 50 AA were further analyzed. In addition, several species showed a potential extended reading frame accessible through an alternative 3′ss. The strength of these 3′ss were analyzed using the MaxEntScan online tool [43]. Extended frames were analyzed for AA composition as described above and compared using standard alignment tools.

### Mass spectrometry

Mouse brain lysates were prepared by homogenizing brain tissue of C57BL/6 WT mice, and single cells were lysed in buffer containing 60 mM Tris pH 7.5, 30 mM NaCl, 1 mM EDTA, and 1x Invitrosol (Invitrogen). Lysates were run on an SDS-PAGE gel and cut into 25 equal-sized Coomassie-stained bands. In-gel digest was performed overnight with 50 ng trypsin (Promega) per band. Digested samples were resuspended in 0.1% (v/v) TFA and 5% (v/v) acetonitrile, and peptides were analyzed by a reversed-phase capillary liquid chromatography system (Ultimate 3000 nanoLC system, Thermo Scientific) connected to an Orbitrap Fusion mass spectrometer (Thermo Scientific). LC separation was performed on an in-house packed 75-μm inner diameter PicoTip column (25 cm) packed with ReproSil-Pur C18AQ particles, 3 μm, 120 Å (Dr. Maisch, Germany). The flow rate was 200 nL/min using gradient of 3–30% B in 60 min. Mobile-phase solvent A contained 0.1% formic acid in water and mobile-phase solvent B contained 0.1% formic acid in acetonitrile.

For standard MS/MS measurements, FT survey scans were acquired with a resolution of 120,000. The data-dependent acquisition (DDA) mode and monoisotopic precursor ions with charge states 2 and 3 were selected. HCD MS/MS spectra were acquired in the linear ion trap using a quadrupole isolation window of 1.6 Da, an AGC target value of 5E3, and a normalized collision energy of 30%.

In data-dependent acquisition (DDA) MS/MS measurements, many of the (often low-intensity) proteotypic frameshift-peptides are masked by mass peaks of peptides belonging to more abundant species. In order to overcome the dynamic range issue and to increase the probability of detection of these peptides, we applied a targeted SIM approach by using multiplexed m/z windows (quadruple mass filtering) corresponding to the targeted mass list of peptides belonging to expected frameshift-peptides of proteins identified in DDA experiments.

For targeted SIM experiments, three 3 FT scans of multiplexed quadrupole selected m/z windows (each 10) of a width of 1.6 Da were measured at a nominal resolution of 120,000 followed by HCD MS/MS scans of recognized targeted precursor ions. The MS/MS spectra were acquired in the linear ion trap (DDA mode) with an AGC target value of 5e3 and normalized collision energy of 30%. In standard DDA measurements, precursor ions with the highest relative intensities (see Fig. 2b, red circles) are chosen for MS2 fragmentation and subsequent identification. In targeted SIM measurements, high-intensity signals outside the specific (targeted) peptide mass are excluded by quadrupole filtering, and thus, peaks may be recognized, fragmented, and further identified due to the increased sensitivity.

Peptide identification for the standard MS/MS measurements was performed using Mascot Distiller (version 2.5.1.0), while the targeted SIM MS/MS data were searched using Mascot Search engine (version 2.5). Data were searched against a custom database including all reviewed, annotated Uniprot mouse entries, plus annotated Uniprot mouse isoform entries and predicted frameshift isoforms. The mass tolerance of precursor and sequence ions was set to 15 ppm and 1.0 Da, respectively, and two missed cleavage sites were allowed. Peptides identified as mapping to frameshift isoforms by the Mascot software were confirmed as proteotypic (i.e., unambiguously identifying only the frameshift isoform) by cross-referencing with the same search database. For full-scan measurements, two brain lysate samples were run on two gel lanes and measured independently (*n* = 2).

### Proline-rich motif analysis

For mouse and human transcripts, canonical C-terminal sequences and predicted frameshift sequences with passing scores were analyzed for frequency of known SH3-domain-binding proline-rich motifs. Frequency scores were calculated based on previously described methods [44, 45]. Briefly, the probability of finding a given motif by chance was calculated by multiplying the frequencies of each AA in the appropriate organism’s annotated Uniprot database by the entire length of the protein database. Then, the number of patterns actually found in each sequence was compared to the number of patterns expected to be found for a sequence of that length. If the number of motifs found is the same number as expected, a frequency score of 1.0 is given. Scores above 1.0 represent overrepresented motifs occurring more frequently than expected by chance, while scores under 1.0 represent underrepresented motifs. If no motifs are found in a given sequence, a frequency score of 0 is given. Custom Python scripts were used to apply this metric to all predicted frameshift C-termini.

### Disorder prediction

The disorder of the frameshift C-termini was approximated by predicting the secondary structural elements of the full-length and frameshift C-terminal sequences using the SPIDER2 algorithm [46]. C-terminal sequences were generated by appending the canonical or frameshift C-termini with the 10 residues located upstream (N-terminally) of the C-terminus, if available in the annotated Uniprot database. The proportion of proline was calculated for full-length and frameshift C-terminal sequences, and the 200 candidates with the largest difference in proline content (with more proline generated in the frameshift C-terminus) were chosen for secondary structure analysis. The number of residues with a “coil” (unstructured) prediction was compared with the entire length of the C-terminal sequence to calculate the %coil score, an approximation of the degree of disorder of each sequence.

### Cell culture, transfections, and treatments

Hek293T cells were cultured in DMEM medium (Biowest) containing 10% FBS (Biochrom) and 1% Pen/Strep (Biowest). Transfections of Hek293T cells using RotiFect (Carl Roth) were performed according to the manufacturer’s instructions. For the measurement of protein stability, CHX (Sigma) was added 48 h after transfection at a final concentration of 40 μg/ml. The proteasome inhibitor MG132 (Biomol) was used at a concentration of 10 mM.

### CRISPR-Cas9

For genome-engineering in Hek293T cells, sequences flanking the 5′ss of PDE6G exon 3 were analyzed for sgRNA candidates in silico. A pair of oligos for the highest ranked candidate sgRNA [47] was synthesized and subcloned into the pSpCas9 (BB)-2A-Puro: PX459 vector (kindly provided by Stefan Mundlos). A repair template was designed to encode homologous arms (60 bp, each) flanking the G>T mutation in the 5′ss of PDE6G, reverse complement sequence: 5′-GTG CTG GGT GTG CCT GGG GGG ACC TGG GCA GAC CTC GGG TTG GTA CTG GCA G**A**C GTC ATA ACT GTT CCC AGG CCT TCC ATT CCA GGG ATG TCG TCC CCA AAC CTG CAA GGA CAGAGCACT-3′ (bold: silent mutation of a BceAI restriction site, underlined: G>T mutation, explanation see below). Hek293T cells were transfected in 24-well plates using Rotifect following the manufacturer’s protocol. For each well of a 24-well plate, a total of 400 ng Cas9+sgRNA plasmid and 500 pmol/μl of the repair template was used. Forty-eight hours after transfection, the transfected cells were selected with 1 μg/ml puromycin and clonal cell lines were isolated by dilution [47]. Genomic DNA was extracted using DNA extraction buffer (200 mM Tris pH 8.3, 500 mM KCl, 5 mM MgCl2, 0.1% gelatin in H_2_O), and a PCR was performed using gene-specific primers (Pde6g_Crispr: forward 5′-GTCCCTGAGTGCTCTGTCCTTG-3′ and reverse 5′-GTGCTGGGTGTGCCTGG-3′). To screen for a successful insertion of the template, a silent mutation was inserted in the template sequence to delete the recognition sequence of an endogenous BceAI restriction site. Removal of the BceAI site upon successful insertion of the template prevents cutting of the PCR product of mutated cells upon digestion with BceAI whereas in wildtype cells the presence of the BceAI site in the PCR product results in fragmentation of the PCR product. Genotypes were confirmed by sequencing. A heterozygous clone carrying the disease-causing point mutation in one allele was further analyzed.

### Constructs

The expression construct for U2af26 and Per1 were previously described [18]. cDNA encoding Pde6g, Meis3, or Clec2l were cloned into the pEGFP-C1 (Clontech) to yield an N-terminally GFP-tagged protein. For U2af26 constructs from mouse, rat, and human, the last exon was amplified from genomic DNA using three different forward primers (resulting in three different ORFs) and a single reverse primer containing the stop codon of the longest frame. The human alternative 3′ss frame was amplified from genomic DNA using matching primers. The prolonged elephant frame was ordered as synthetic DNA (MWG-Biotech). All frames were cloned/shuttled into the pEGFP-C1 vector resulting in N-terminally GFP-tagged constructs. To analyze a potential destabilizing effect on Per1, instable frames of rat and human were fused to the mouse U2af26 N-terminus, which is sufficient to interact with Per1 [18]. To design a codon-optimized frameshift variant, optimal codons for each AA were determined based on the graphical codon usage analyzer [48]. A synthetic DNA oligonucleotide was purchased from MWG-Biotech and designed with restriction sites to directly ligate into the pEGFP-C1 vector (Clontech). To design a proline-free U2af26 frameshift variant, all proline residues were substituted with alanine. Synthetic DNA oligonucleotides were designed with restriction sites to directly ligate them into the pEGFP-C1 vector (Clontech) and were purchased from MWG-Biotech. The human PDE6A and PDE6B constructs were purchased from OriGene. Cloning primers are available upon request. All constructs were verified by sequencing.

### RNA, RT-PCR, qRT-PCR

Total RNA was extracted from mouse tissues or Hek293T cells to analyze gene expression by quantitative RT–PCR. These procedures were performed as previously described [18]. Phosphorimager quantification was done as previously described [18]. The following primer pairs were used to perform a splicing sensitive PCR of mouse genes: Pde6g: forward 5′-GTTTAAGCAGCGGCAAACAAGGC-3′ and reverse 5′-CAGGGCTCACATAGCAGGGATC-3′; Meis3: forward 5′-GCCCATGGCAGGCTTCAC-3′ and reverse 5′-GGGAGCTTTGGAGGTGAAGTCC-3′; Clec2l: forward 5′-CGCTGGCCGTGATCCAAAGC-3` and reverse 5′-GAGCCTCATCACGTATAGGCCATC-3′; Sulf1: forward 5′-GGTATAAACAGTGCA ACCCAAGACCC-3′ and reverse 5′-CCTGTACTCATCGATGTTGCTTGCAC-3′. The primers bind exonic sequences flanking the penultimate exon. Hence, the splicing-sensitive PCR results in a shorter mRNA product if the exon is skipped (resulting in the translation of a frameshift protein, fs) and a longer product if the exon is included (resulting in the translation of the full-length protein, fl). The following primer pair was used to determine the mRNA expression of GFP-tagged constructs using q-RT-PCR: GFP: forward 5′-GAAGCGCGATCACATGGT-3′ and reverse 5′-CCAT GCCGAGAGTGATCC-3′. GAPDH was used as reference gene: GAPDH: forward 5′-CTTCGCTCTCTGCTCCTCCTGTTCG-3′ and reverse 5′-ACCAGGCGCCCAAT ACGACCAAAT-3′.

### Immunoblotting, immunoprecipitation (IP), IP-MS, and antibodies

Cells were lysed in buffer containing 60 mM Tris pH 7.5, 30 mM NaCl, 1 mM EDTA, and 1% TritionX-100. SDS-PAGE and immunoblotting were done according to standard protocols. For IPs, Hek293T cells were transfected as described above and transfection conditions were optimized to result in equal expression levels of N-terminally GFP-tagged PDE6G-fl and PDE6G-fs. Transfected cells were lysed in lysis buffer (60 mM TrisHCl pH 7.5, 30 mM NaCl, 1 mM EDTA, 1% Triton X-100, with protease inhibitors); 100 μg of lysate was incubated in 400 μl RIPA buffer containing 400 mM NaCl and 2% BSA. After 1 h precleaning with A/G beads (Thermofisher) at 4 °C, prewashed anti-FlagM2 beads (Sigma) were added and 4 °C rotation was continued overnight. Beads were washed 4 times in RIPA buffer (10 mM Tris-HCl pH 7.5, 100 mM NaCl, 2 mM EDTA, 1% NP-40, with protease inhibitors), and after the last wash SDS sample buffer was added, samples were boiled and analyzed by SDS-PAGE and immunoblotting.

For IPs of U2af26 fl and fs, cells were harvested and lysed in lysis buffer containing 10 mM Tris-HCl pH 7.4, 150 mM NaCl, 0.5 mM EDTA, 1 mM PMSF, Roche complete protease inhibitor cocktail, Invitrosol (Invitrogen), DNase (NEB), and RNase (Applichem). Protein concentration was measured via Bradford assay, and 3 mg lysate was incubated with 30 μl magnetic GFP-Trap beads (ChromoTek) for 1 h at 4 °C. Beads were washed twice with buffer containing 10 mM Tris-HCl pH 7.4, 150 mM NaCl, and 0.5 mM EDTA and once with TBS. ^16^O/^18^O-labeling was performed to quantify via mass spectrometry. Briefly, U2af26-GFP samples were “heavy” labeled by washing with TBS prepared in H_2_^18^O (Campro Scientific) and performing on-bead tryptic digestion by incubating with buffer containing 2 M urea, 50 mM Tris-HCl pH 7.4 and 5 μg/ml trypsin (Roche) overnight. The following day, peptides were treated with 1 mM DTT and 5 mg/ml iodoacetamide (prepared in H_2_^18^O) according to standard procedures, and trypsin was inactivated with 0.1% TFA. GFP controls were “light” labeled by identical treatment, with all solutions prepared using “light” H_2_^16^O. After inactivation of trypsin, heavy and light samples were mixed in a 1:1 ratio and desalted via Empore C_18_ stage-tips. Both immunoprecipitations were performed in duplicate. Peptides were fractionated in the presence of 0.1% formic acid via reversed-phase chromatographic separation with a Dionex Ultimate 3000 nanoLC (Thermo Fisher Scientific), using a 90-min acetonitrile gradient of 5–60% and a flow rate of 350 nl/min on a self-packed 25 cm silica microcolumn (i.d. 100 μm) packed with ReproSil-Pur C18-AQ 3 μm resin (Dr Maisch GmbH). Eluted peptides were analyzed with an LTQ Orbitrap Velos mass spectrometer (Thermo Fisher Scientific). Proteins were identified and quantified using Mascot Distiller software (version 2.5.1.0) and searched against the human Uniprot database, to which the mouse U2af26 isoforms and RNAseq-confirmed human frameshift isoforms had been appended. Heavy/light intensity ratios were normalized by dividing all values with the median ratio. Proteins quantified with a heavy/light ratio greater than 1.5 in both replicates were considered enriched, representing interaction partner candidates. Tubulins and HSPs were not considered as they represent likely false positives.

Antibodies used for immunoblotting: GFP (Santa Cruz, B-2), α-GAPDH (GeneTex, GT239), α-hnRNPL (Santa Cruz, sc-32317), Vinculin (Santa Cruz, sc-5573), PDE6G (Santa Cruz, sc-98,466), and α-FLAG (Cell Signaling, 2368). Immunoblots were quantified using the GelQuant.NET software provided by biochemlabsolutions.com.

### Statistical analysis

Quantifications represent mean values of at least 3 independent experiments (exact numbers are given in the figure legends), and error bars represent standard deviation. Significance was calculated by Student’s unpaired *t* test: *p < 0.05, **p < 0.01, ***p < 0.001.

## Supplementary information

**Additional file 1: Figure S1.** Skipping of penultimate exons is regulated in a tissue- and developmental stage-specific manner. **Figure S2.** Alternative C-termini display reduced basal expression and fast proteasomal degradation, whereas mRNA levels are not reduced. **Figure S3.** A codon-optimized U2af26 alternative C-terminus displays increased basal expression and unchanged proteasomal degradation. **Figure S4.** Skipping of the penultimate exon in human candidates is regulated in a tissue-specific manner and conserved between mouse and human. **Figure S5.** Comparison of U2af26 frames from different mammalian species. **Figure S6.** Prolonged U2af26 C-termini are unstable through proteasomal degradation. **Figure S7.** Prolonged U2af26 C-termini from different species destabilize Per1.

**Additional file 2: Table S1.** List of mouse and human candidates with extended frames in the 3’UTR as well as conservation of frameshift-inducing alternative splicing of the penultimate exon between mouse and human.

**Additional file 3: Table S2.** Frameshift peptides identified by mass spectrometric analysis of mouse brain lysate and PxxP motif frequency in mouse and human candidates.

**Additional file 4: Table S3.** Correlation of protein half-life and proline content of frameshift-proteins.

**Additional file 5: Table S4.** Potential extended frames allowing translation into the 3’UTR of all mammalian species harboring a U2af26 gene.

**Additional file 6: Table S5.** Interactions of U2AF26fl and U2AF26fs analyzed by mass spec.

**Additional file 7.** Review history.

## Data Availability

All RNA-Seq data used in the present study is publicly available. RNA-seq data of 22 mouse tissues were downloaded from the mouse ENCODE project (accession number: SRP012040, https://www.ncbi.nlm.nih.gov/geo/query/acc.cgi?acc=GSE36025). RNA-seq data of 16 human tissues were downloaded from the Illumina Human Body Map 2.0 project (accession number: ERP000546, https://www.ncbi.nlm.nih.gov/geo/query/acc.cgi?acc=GSE30611). RNA-seq data from mouse 3T3 cells (accession number: ERR498282 and ERR498284 [28];) and data generated across a time series of differentiation of cortical glutamatergic neurons from murine embryonic stem cells (accession number: SRP017778 [49];) were downloaded from previous studies. All other material is available upon request to F.H. (florian.heyd@fu-berlin.de).
