# Supplementary material for: Splicing-accessible coding 3′UTRs control protein stability and interaction networks
Source: Genome Biol. 2020 Jul 29;21:186. doi: 10.1186/s13059-020-02102-3 (PMC7392665; doi:10.1186/s13059-020-02102-3)
Supplement: Supplementary file 1 — Additional file 1: Figure S1. Skipping of penultimate exons is regulated in a tissue- and developmental stage-specific manner. Figure S2. Alternative C-termini display reduced basal expression and fast proteasomal degradation, whereas mRNA levels are not reduced. Figure S3. A codon-optimized U2af26 alternative C-terminus displays increased basal expression and unchanged proteasomal degradation. Figure S4. Skipping of the penultimate exon in human candidates is regulated in a tissue-specific manner and conserved between mouse and human. Figure S5. Comparison of U2af26 frames from different mammalian species. Figure S6. Prolonged U2af26 C-termini are unstable through proteasomal degradation. Figure S7. Prolonged U2af26 C-termini from different species destabilize Per1. [file 13059_2020_2102_MOESM1_ESM.pdf]

Splicing-accessible coding 3'UTRs control protein stability and interaction networks

Marco Preussner[1] ≠, Qingsong Gao[2] ≠, Eliot Morrison[3] ≠, Olga Herdt[1], Florian Finkernagel[4], Michael Schumann[5], Eberhard Krause[5], Christian Freund[3], Wei Chen[6]*, Florian Heyd[1]*

Supplementary Information

Figure S1: Skipping of penultimate exons is regulated in a tissue- and developmental stage-specific manner.

A) Schematic representation of the bioinformatics prediction pipeline identifying 3233 transcripts with ≥20 new AA and ≥10 AA past the canonical stop codon. **B)** Barplot showing the number of penultimate exon skipping events found across different mouse tissues using RNA-seq data; position of the stop codon of the respective transcript is indicated (ultimate or penultimate). **C)** Barplot showing the number of penultimate exon skipping events across different tissues with the number of different tissues supporting the skipping event. This analysis identifies tissue-specific and pan-tissue events. **D)** Barplot as in B but looking at neuronal differentiation. **E)** Barplot showing two examples with consistently increased/decreased PSI values during differentiation (*Dock9* and *Itga3* gene in top and bottom panel, respectively). **F)** Barplot showing percentage of exons with supporting RNA-seq for internal frame-preserving (Internal_fp), internal frameshifting (Internal_fs) and penultimate exon that are either frame-preserving (Penulti_fp), or frame shifting leading to a an extended C-terminus with at least 20 alernative amino acids (Penulti_long) or a shorter alternative C-terminus (Penulti_short) using different cutoffs for an exon to be considered alternative. **G**) Motif analysis for penultimate frameshift-inducing exons that are alternatively spliced during neuronal differentiation with a ∆PSI of 50% between any two stages. We used the sequences of these penultimate exons as well as their 50bp upstream and downstream introns as input for Homer search (see Material and Methods for details). Two significantly enriched motifs are shown on top and annotated motifs are shown below.

H) Expression of the two RBPs during neuronal differentiation whose binding motifs were found to be enriched in regulated penultimate exons in G).

Figure S2: Alternative C-termini display reduced basal expression and fast proteasomal degradation, whereas mRNA levels are not reduced.

A-D) Hek293T cells were transfected with N-terminally GFP-tagged full-length constructs of U2af26 and Pde6g or the N-terminally GFP-tagged C-termini of Meis3 or Clec2l. Protein stability and proteasomal degradation was determined by Western blot and the addition of CHX and/or MG132. Shown are representative blots and quantifications (mean +/- SD) from n=3. Schematic representation of the transfected constructs are shown for Pde6g, Meis3 and Clec2 (right). **E)** Relative mRNA expression of Pde6G, Meis3, and Clec2l in Hek293T cells transfected with the respective GFP-tagged constructs, n=3.

Figure S3: A codon-optimized U2af26 alternative C-terminus displays increased basal expression and unchanged proteasomal degradation.

A) Hek293 cells were transfected with N-terminally GFP-tagged U2af26-fs encoding a wildtype (fs) or a codon optimized variant (OPT) of the frameshift sequence and basal expression levels were determined 48 hours later by immunoblotting, n=4. **B)** Hek293T cells were transfected as in A) and protein stability was determined as in Figure 3C. For visualization, a shorter exposure time of U2af26-fs-OPT is shown.

Figure S4: Skipping of the penultimate exon in human candidates is regulated in a tissue-specific manner and conserved between mouse and human.

A, B) Barplot showing the number of penultimate exon skipping events across different human tissues using RNA-seq data as in Supplementary Figure 1B and 1C. **C**) Strongest pathway enrichments for genes with penultimate exon skipping events conserved between mouse and human. **D)** Identification of human-mouse conserved penultimate exon skipping events for exons where skipping leads to a shorter C-terminus (top) or for frame-preserving exons (bottom). The same pipeline as in Figure 4B was used and the respective numbers are shown. **E**) Mean % coiled for human C-termini comparing fl and fs versions as in Fig 3B.

Figure S5: Comparison of U2af26 frames from different mammalian species.

A) Conserved alternative 3’ splice sites (ss) in the last U2af26 exon. Alignment of alternative 3’ss from all species that give access to a potential 3’UTR-encoded protein-isoform with more than 50 AA. On the right the MaxEnt 3’ss splice site score for the underlined sequences is depicted. **B)** Alignment of long U2af26 C-termini from diverse species with experimentally confirmed low half-life time. -1 frames from mouse, rat, and elephant show more similarities than the +1 human frame or the human frame accessible through an alternative 3’ss. Generally, there is no conservation or common motifs. **C)** AA composition of extended U2af26 C-termini. The percentage of each AA for the 58 prolonged C-termini (>50 AA) is shown relative to the average abundance in all proteins (UniProt/SwissProt). **D)** Stability of the hypothetical short alternative mouse +1 frame determined as in Figure 3C. n=3.

Figure S6: Prolonged U2af26 C-termini are unstable through proteasomal degradation.

A-D) The stability of rat, human and elephant frames was determined as in Supplementary Figure 2 and experiments were repeated in the presence of the proteasome inhibitor MG132. Shown are representative blots and quantification mean +/- SD (n>3).

Figure S7: Prolonged U2af26 C-termini from different species destabilize Per1.

A) Scheme illustrating GFP-tagged U2af26 constructs encoding alternative C-termini of different species that were designed to address their ability to destabilize Per1. The constitutive mouse U2af26 N-terminus is sufficient for interaction with mouse Per1, through binding to the PasB domain, and was fused to prolonged C-termini from rat and human. **B)** Both, the alternative rat frame and the human frame, which is accessible through an alternative 3’ss, destabilize co-transfected Per1 (here compared to the constitutive mouse C-terminus). Hek293T cells were co-transfected with the N-terminally GFP-tagged U2af26 frames from different species and Per1-FLAG, treated as indicated, and protein stability was determined as in Figure 3C, (n=4).

Table S1: List of mouse and human candidates with extended frames in the 3’UTR as well as conservation of frameshift-inducing alternative splicing of the penultimate exon between mouse and human.

Table S2: Frameshift peptides identified by mass spectrometric analysis of mouse brain lysate and PxxP motif frequency in mouse and human candidates.

Table S3: Correlation of protein half-life and proline content of frameshift-proteins.

Table S4: Potential extended frames allowing translation into the 3’UTR of all mammalian species harboring a U2af26 gene.

Table S5: Interactions of U2AF26fl and U2AF26fs analyzed by mass spec.

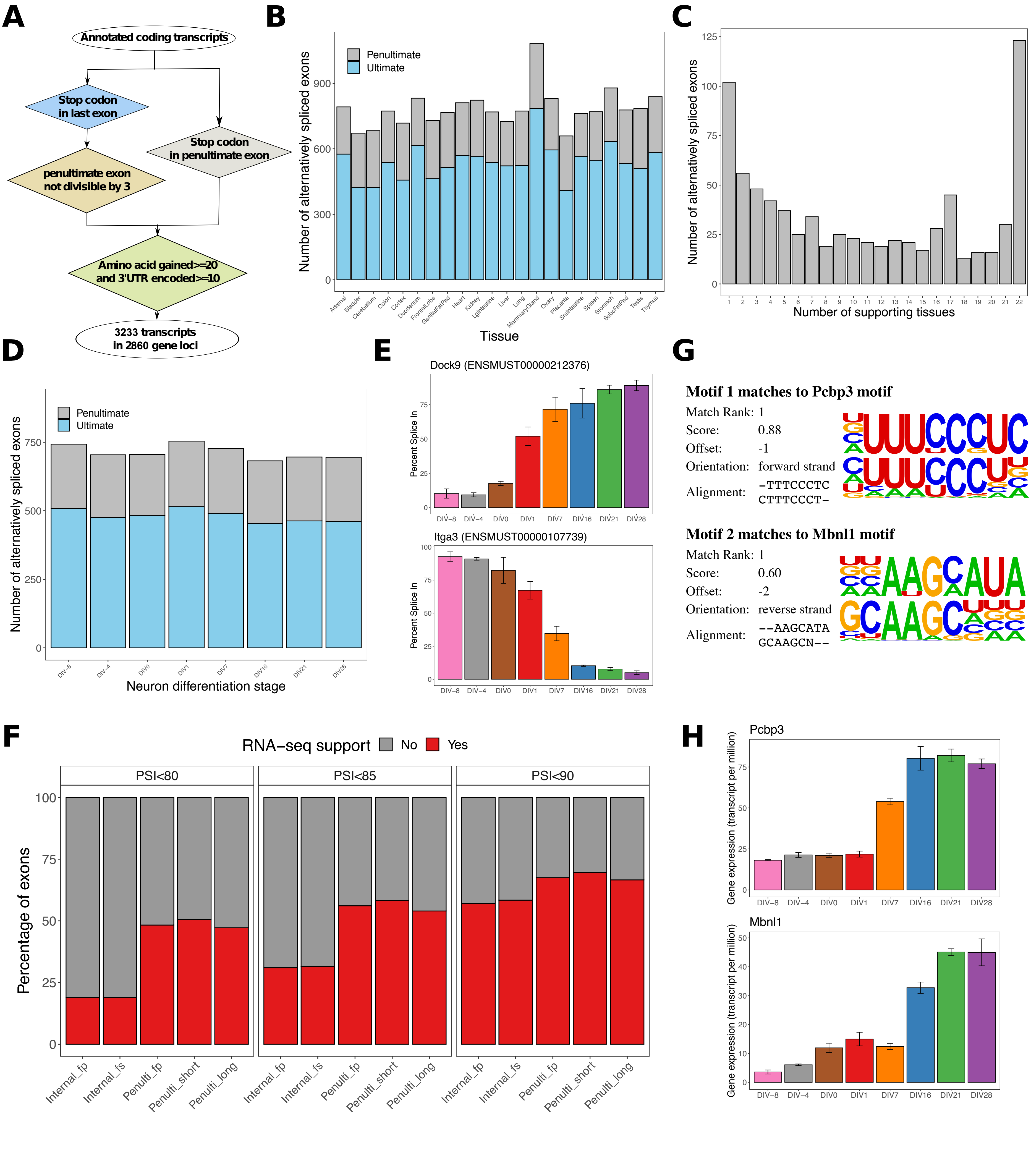

Preussner et al., Supp. Fig. 1

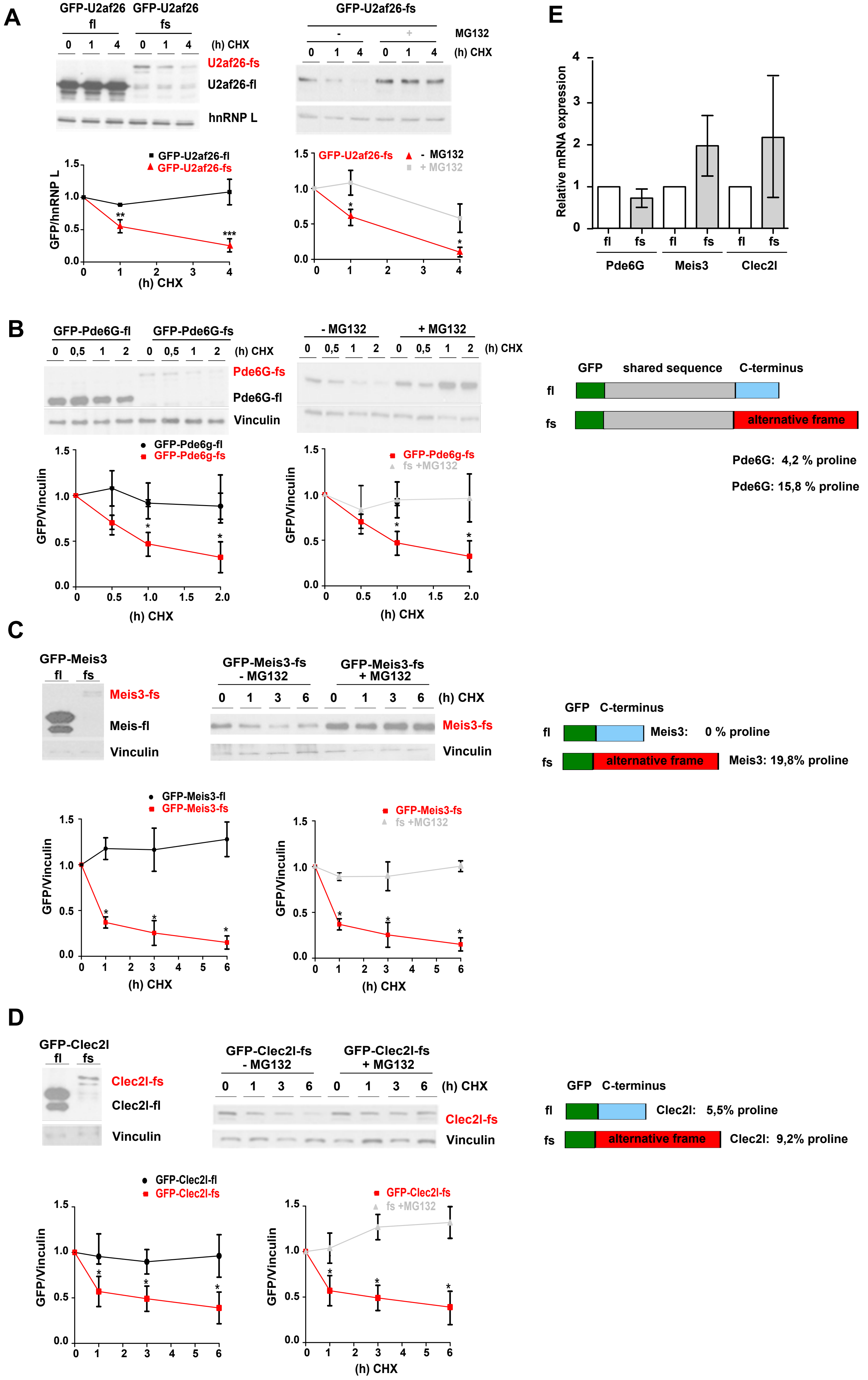

Preussner et al., Supp. Fig. 2

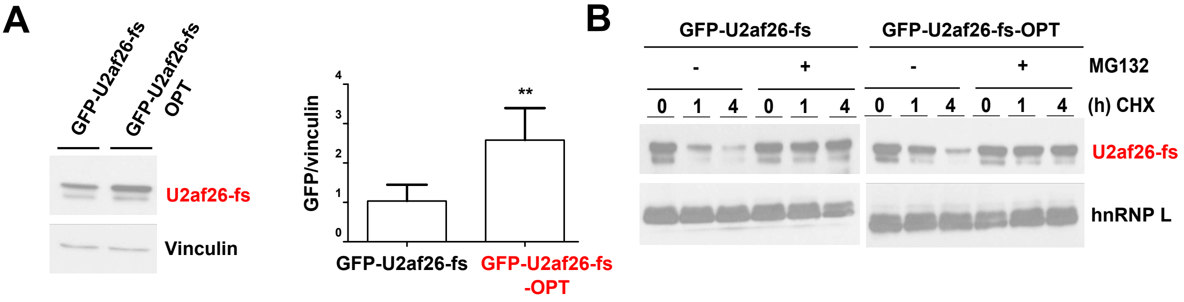
A
GFP-U2af26-fs
GFP-U2af26-fs OPT
U2af26-fs
Vinculin
GFP/vinculin
4
3
2
1
0
**
GFP-U2af26-fs
GFP-U2af26-fs -OPT
B
GFP-U2af26-fs
GFP-U2af26-fs-OPT
MG132
-
+
-
+
(h) CHX
0
1
4
0
1
4
0
1
4
0
1
4
U2af26-fs
hnRNP L

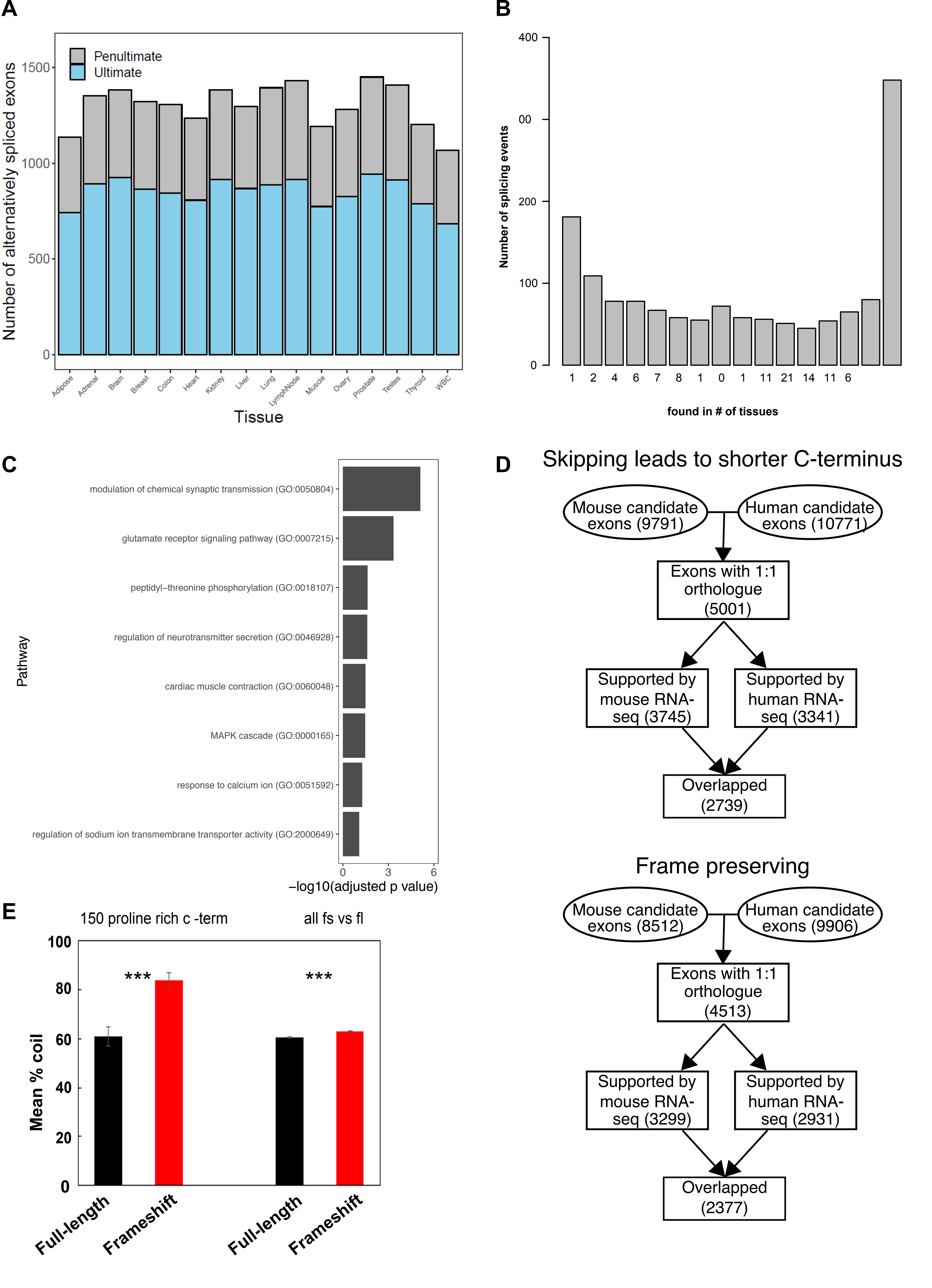

Preussner et al., Supp. Fig. 4

A

```
                                       alt. 3'ss                                                        3'ss score
CCTCAAAGCCCCCTTCACTCTCCTGCCCCATCCT-TCCCAGGCTCCCGGGCTCCATAATG   chimp (P.troglodytes)                 7.7
CCTCAAAGCCCCCTTCACTCTCCTGCCCCATCCT-TCCCAGGCTCCCGGGCTCCATAATG   human (H.sapiens)                     7.7
CCTCAAAGCCCCCTTCACTCTCCTGCCCCATCCT-TCCCAGGCTCCCGGGCTCCATAATG   gorilla (G.gorilla gorilla)           7.7
CCTCAAAGCCCCCTTCACTCTCCTGCCCCATCCT-TCCCAGGCTCCCGGGCTCCATAATG   orangutan (P.abelii)                  7.7
CCTCAAAGCCCCCTTCACTCTCCTGCCCCATCCT-TCCCAGGCTCCTGGGCTCCATAATG   gibbon (N.leucogenys)                 7.7
CCTCAAAGCCCCCTTCACTCTCCTGGCCCATCTT-TCCCAGGCTCCTGGGCTCCATAATG   rhesus (M.mulatta)                    6.6
CCTCAAAGCCCCCTTCACTCTCCTGGCCCATCTT-TCCCAGGCTCCCGGGCTCCATAATG   baboon (P.anubis)                     6.6
CCTCAAAGCCCCCTTCACTTTCCTGCCCTGTCTT-TCCCAGGCTCCCGGGCTTCATAATG   squirrel monkey (S.boliviensis)       10.3
CCTGAAAGCTCCCTTACCTCGCCAGCGCCGTCTT-CCCCAGACTCCGGGGCTCCAAGATG   dog (C.lupus familiaris)              6.3
CCTGAAAGCTCCCTTAACCCCCCAGCTCCATCTTCCCCCAGGCTCCGGGGCTCCATGCTG   panda (A. malanoleuca)                8.5
TCTCAAAGTCCTCTTCACTTTCCAGCTCTACCAT-CCCCAGGTTCTGGGGCTCCATAATG   mouse lemur (M.murinus)               6.6
 ** ****  * ***..*   **:*  *  . *  *  *****. **  ***** **:..**
```

B

```
human_fs+1        ---------------------HPRGSILATIP---------------------------
human_alt3'ss     -----------------------------------------------------------
elefant_fs-1      VAPEVSYWPPSPREEPTAFARPPAWPLLKPWPPC---------PLPLTGTVPGLTPPQSP
mouse_fs-1        VTSKVPHRSPSPRKEPTSFPRPPAWSLLRRVPLLSTQGHRCSCPASLFKVPFTLQ-SQHP
rat_fs-1          VTSKVPYRSPTPRKEPTPFPRPPAWSLLRLVPLLFTQGHRCSCLASLLKALYTFQ-SHHP

human_fs+1        ERGTIGVPLITG-----MAASEALAPLPFTPNRDRCSWQDLSSKPPSLSCPILP------
human_alt3'ss     -------------------APG-LH------NVICSAWRPSSTAP--V---LIKLRVSLR
elefant_fs-1      SRPTLIFPRLRNSAVKSVGRRD-LI------KYVFVSLLPGVSSP--PPCLLTEGEVRLG
mouse_fs-1        Q------------------ASE-LH------NIICST---QEPPP--PVPPPIKVKKFVN
rat_fs-1          Q------------------ASE-LH------NTMFDT---GTSSS--SFCPSSNKA----
                                         *       :     :

human_fs+1        ------------------------------------------------------------
human_alt3'ss     HQLAFAVVWHFPPPTHVTKRGNKTGSWPQLQLRNLARPAWDPGP----------------
elefant_fs-1      SGQNLS---------HR--------ATSHLTGGEAAGAGRGPGGP---------------
mouse_fs-1        QGLWFPS---TPTPKKG--------CKPTTVSAEELGASLGSGSALVSRGCFNTCNLRLS
rat_fs-1          ------------------------------------------------------------

human_fs+1        -----
human_alt3'ss     -----
elefant_fs-1      -----
mouse_fs-1        ILGAF
rat_fs-1          -----
```

C

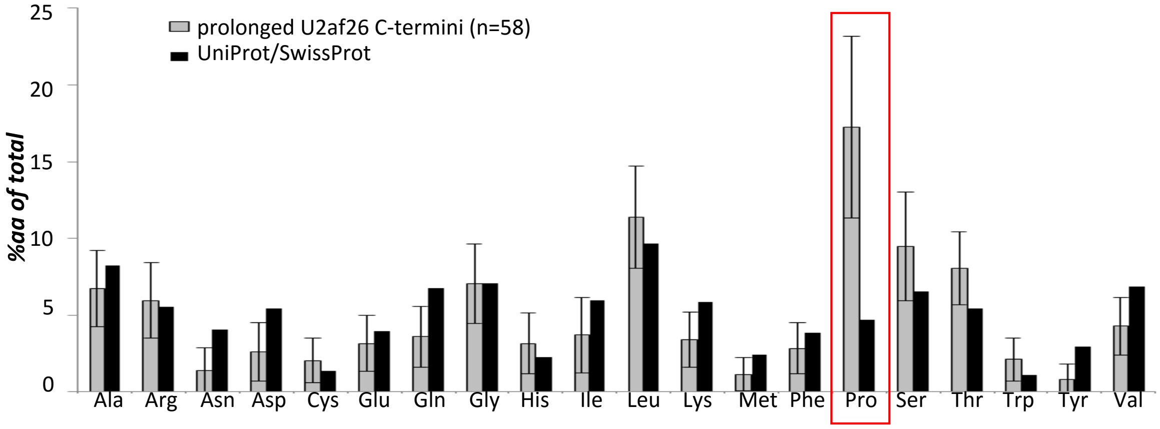

D

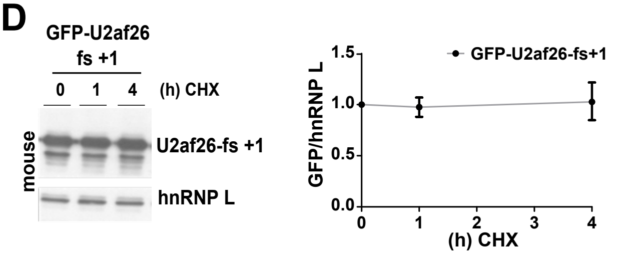

Preussner et al., Supp. Fig. 5

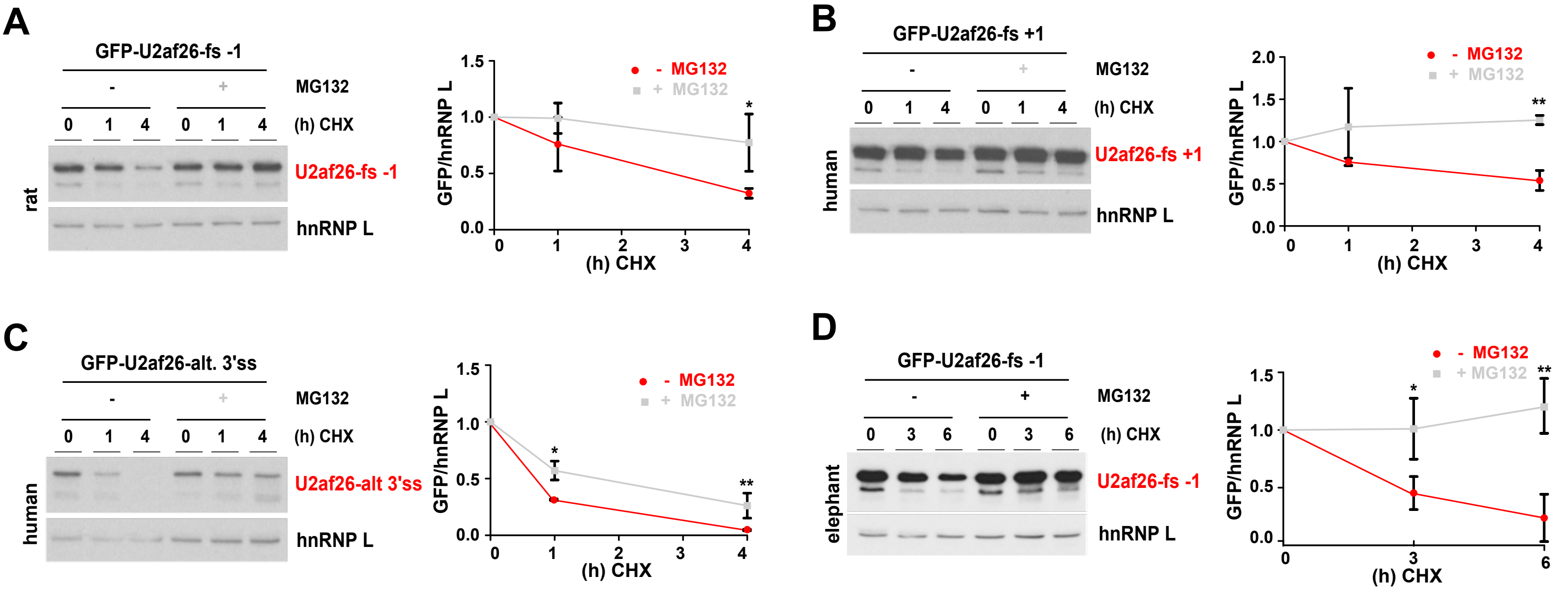

Preussner et al., Supp. Fig. 6

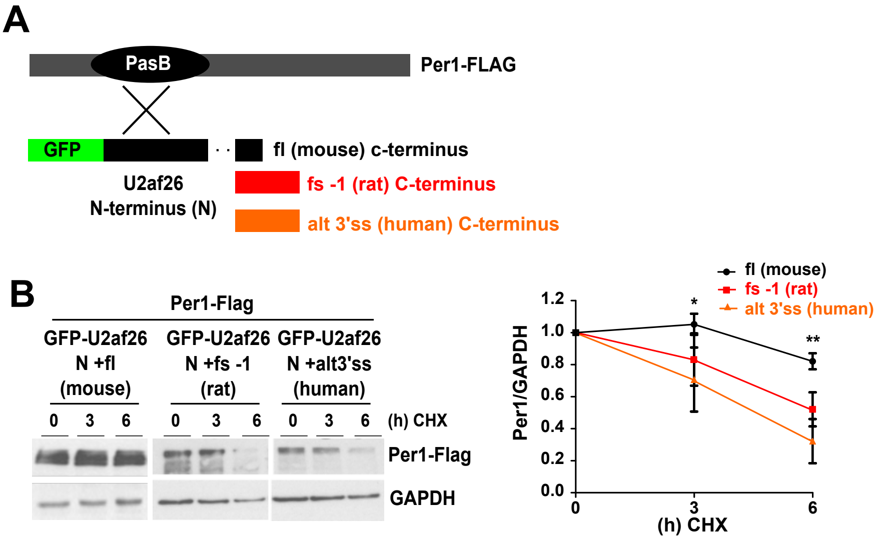

Preussner et al., Supp. Fig. 7
