# Supplementary material for: Splicing-accessible coding 3′UTRs control protein stability and interaction networks
Source: Genome Biol. 2020 Jul 29;21:186. doi: 10.1186/s13059-020-02102-3 (PMC7392665; doi:10.1186/s13059-020-02102-3)
Supplement: Supplementary file 7 — Additional file 7. Review history. [file 13059_2020_2102_MOESM7_ESM.docx]

Review History

**First round of review**

**Reviewer 1**

**Are you able to assess all statistics in the manuscript, including the appropriateness of statistical tests used?**

Yes, and I have assessed the statistics in my report.

**Comments to author:**

Preussner and colleagues present a very interesting and original study describing how skipping of penultimate exons in mammals can extend the C-termini by creating a frame shift that leads to translation into the 3′ UTR. The authors report that at least 10% of human and mouse genes have a penultimate exon that is skipped significantly (as judged by RNA-seq) and that extends the C-termini by at least 20 aminoacids. Then, they show that these extended sequences are enriched for disordered regions and PxxP motifs, which can abolish or rewire protein-protein interactions and lead to decreased protein half-life. The authors suggest this may be a regulatory mechanism for some genes, but also a control mechanism for others, since skipping of penultimate exons does not lead to NMD. Finally, they exemplify their results by focusing on the cases of U2af26 and PDE6G. In general, I believe this is a nice study worth of Genome Biology. However, I have a few comments/concerns:

1) One conceptual issue that requires further clarification and analysis is the very idea of "extended translation into 3′ UTRs upon skipping". It was not clear to me how often this is due to skipping in the canonical isoform, as the manuscript seems to implicitly suggest, or due to skipping of an "alternative", minor, penultimate exon. In other words: is it possible that in many cases they are looking at penultimate exons that are rarely included and, when they are included, they lead to shorter C-termini? I think it would be important to show how often their penultimate exons are part of major or minor isoforms (e.g. by showing the average PSI of the exon). Along these lines, they show the fraction of exons with evidence for skipping by RNA-seq (e.g. ~50% for PSI < 80), but it would be important to also show the fraction of them with significant inclusion (e.g. with PSI > 50 and >90, etc).

2) Relatedly, it would be interesting to see the numbers for all types of penultimate exons genome-wide. They currently focus on a specific type of penultimate exon, but it is not clear how other penultimate exons behave. Namely: A) penultimate exons that are multiple of three; A.1) penultimate exons that are not multiple of three and lead to shorter C-termini when skipped; and A.2) penultimate exons that are not multiple of three and lead to longer C-termini when skipped (their set of interest). It will be important to compare these three sets in terms of PSIs, conservation, enrichment of PxxP motifs, etc. instead of just comparing their set of penultimate exons to internal exons (e.g. Figure 1E).

3) The evolutionary conservation of their penultimate exons based on liftover seems quite low (950 have 1-to-1 orthologous between human and mouse out of 3233 mouse exons, or 29%; Figure 4). This value is typical for alternative exons, but not for "major", highly included exons (see point 1). This is, of course, not a problem per se, and it is still remarkable that the majority of those 1-to-1 orthologs are alternative in both species, but the authors should acknowledge the relatively low level of conservation at the orthologous level and how it compares to the other types of penultimate exons (point 2).

4) I think Figures 5F and 5G could be more informative. I agree they show dramatic changes, but it is not obvious what is changing. It would be good to complement it with bar plots for the main interactors in the two isoforms and a few of the "novel" interactors.

5) Page 15: The authors remove exons that share all start and end positions to avoid multiple counting. However, I do not understand why they count multiple times the same exon if the upstream and downstream exons are different. I think it would be more appropriate to count the actual exons only once, even if they come in different combinations.

6) I have some minor/formal comments about U2AF26. First, its official gene symbol should be provided. Second, assuming it is U2AF1L4, I could not find which was the frame-shifting penultimate exon they are discussing, since the length of the main U2AF1L4's penultimate exon is multiple of three. Looking into their original U2af26 paper, I saw that they might be referring to skipping of the second and third to last exons together. If correct, please acknowledge this, at least in the Figure legend. Otherwise, perhaps clarify the issue.

**Reviewer 2**

**Are you able to assess all statistics in the manuscript, including the appropriateness of statistical tests used?**

Yes, and I have assessed the statistics in my report.

**Comments to author:**

In the current manuscript, Preussner et al are providing an evidence for the coding potential of 3' UTR in human cells. Using the computational approach, they predict skipping of the penultimate exons by alternative splicing that leads to the frame shift and extension of mRNA coding potential in the areas of 3' UTR beyond the translation termination stop codon. Such events can stay undetected by the nonsense mediated decay machinery and lead to the production of the proteins with novel C-termini that differ from currently annotated isoforms. Authors have validated the presence of the peptides that can arise in such a manner using mass spectrometry of the mouse brain tissue. This is an exciting observation, since such a peptides are frequently overlooked/masked during the mass spectrometry experiments. Amino acid sequence analysis of "translate through" proteins is enriched in proline-rich motifs, both in mouse and human samples, which suggests their role in mediating protein-protein interaction.

Expression of 3' UTR derived portions of protein sequences fused with GFP exposed their reduced stability, likely through increased proteasome degradation rate. In addition, changes in C-terminal sequence results in the rewiring of the protein-protein interaction network for some of the investigated examples (like U2AF26) which can have a potential downstream effect on the protein targets.

Using CRIPSR/Cas9 authors have recapitulated a previously reported mutation in the exon 3 of PDE6G that leads to the usage of cryptic 5' splice site and "translate through" canonical stop codon. This alteration does not affect the expression level of the mutant allele on the RNA level that stayed comparable to the wild type. At the same time, however, the drastic effect is observed on the protein level. The amount of protein appears greatly reduced potentially due to the destabilization by proteasome. Co-immunoprecipitation of the C'- term. extended protein suggests a complete loss of the interaction with another subunits of the complex.

Mechanism proposed here points towards the idea of unstable protein production due to the high proteasome degradation rate of PDE6G. Homozygous mutation observed in patients in this scenario would lead to almost complete loss of the functional and stable PDE6G that is a necessary component of the protein complex, promoting rapid and early retinal degeneration.

I believe this work would be interested to the wide audience of biologists

I have several comments regarding the current work:

1. Brain tissue is the most enriched in the alternative splicing events that drastically and dynamically change throughout the development, is it possible that the high detection rate of extended proteins in this tissue is simply correlated with the high alternative splicing activity and large amount of alternatively spliced exons?

2. Despite relatively high observation rate (10% reported here) of the 3' UTR translation the evidence of the molecular and biological functionality is still vague. High degradation rates, reduced high-life time is suggestive of the quality control (fail safe) mechanisms. Would be great to add this to the discussion and dial down the functional role of the 3' UTR coding.

3. Did you find examples where the C' terminal extension encoded for a known protein domain or at least parts of it?

4. Do these rather unstructured and flexible proteins parts affect the phase separation properties? Would be great to predict it computationally using some of the recently published tools. For example with: https://doi.org/10.1101/842336, catGRANULE or any others.

5. What are the go-terms associated with the identified the mRNAs that have 3' UTR coding potential? For instance, AS in neurons was recently shown to be enriched in genes coding for ion channels. In addition, AS exons can be regulated by neuronal activity itself (Gonatopoulos-Pournatzis T., Mol. Cell 2020).

6. The regulation of AS in the CNS is relatively well characterized and key regulators of multiple alternative exons are known. These RBPs also exhibit a dynamic expression pattern at different stage of neuron development and maturation (Weyn-Vanhentenryck, 2018, Nature Com). Is there an enrichment for certain RBP targets in the catalog of mRNAs described here that are associated with penultimate exon skipping? This can help to understand if such a skipping is an aberrant event or rather functionally regulated mechanism.

7. Is G/T a common mutation in RP? Can be mentioned in the discussion.

Minor comments:

1. What is the protein expressed in the control lanes as a FLAG fusion in figure 6E. Needs to be better described in the figure legend.

2. Also, the co-IP's (figure 5F and G) are not presented in a visually appealing way, which makes them less intuitive. Please modify and label the top protein partners that have been lost/gained due to the C-terminus modification.

**Reviewer 3**

**Are you able to assess all statistics in the manuscript, including the appropriateness of statistical tests used?**

No, I do not feel adequately qualified to assess the statistics.

**Comments to author:**

In this work, Preussner and colleagues present a thorough report of a conserved expansion of the protein-coding potential of 3΄UTRs in mouse and human cells. Following a bioinformatic analysis that reveals alternative splicing as a common mechanism to extend ORFs in the 3΄UTRs, they observe and biochemically characterize an increase of C-terminally-extended proline-rich motifs that can reduce the protein stability of the corresponding isoforms and in the case of U2af26 they show a profound impact on its interactome. Last, they relate this phenomenon with a retinitis pigmentosa-causing mutation, highlighting the clinical significance of these findings. Overall, this is a work of broad significant interest, the experiments are conducted carefully and convincingly to provide a thorough characterization of this phenomenon and the manuscript is easy to follow. After addressing the points below, I judge this manuscript suitable for publication.

Major points

1. Results, p. 9: Concerning the verification of expression of the 3΄UTR-encoded variants on protein level: Is there a reason why a mass spectrometry analysis was not also performed in human cells? It would be advisable to verify the ORF extension in the 3΄UTR in the same way as in mice. This would strengthen the biological relevance of the phenomenon and would allow a more direct comparison with the results from mouse cells.

Additional points

1. Results, p.5, lines 5-12: Authors convey the notion that protein-coding genes are not expected to contain untranslated exons in their 3΄UTRs. Introns in the 3΄UTR of a gene are indeed expected to stimulate NMD through the deposition of an exon-junction complex (EJC) during splicing of the corresponding mRNA. However, even though a splicing event in the 3΄UTR is expected to trigger NMD, this does not mean that the entire population of this mRNA is rapidly degraded. The sensitivity of a transcript population to NMD varies, depending on several factors (number of splice junctions and length of the 3΄UTR, the presence of uORFs, etc.) and there are examples of protein-coding mRNAs with a termination codon in penultimate exons (such as human CALM3 - calmodulin 3 mRNA). I suggest editing this segment accordingly to consider this point.

2. Results, p.6, lines 1-2: Since the results of this work show that extending the coding potential of the 3΄UTR is conserved it could be that some of these protein isoforms may have biological significance and therefore are not necessarily degraded from the cell. Taking this, the sentence of p.8 lines, 32-33 and parts of the discussion (e.g. p.13, lines 24-25) into consideration, I propose rephrasing this sentence accordingly.

3. Results, p.6, lines 5-7: To address this question, direct evidence on the bulk translation of these mRNAs would need to be provided (e.g. Ribosome profiling evidence). Therefore, a broader re-definition of 3΄UTRs based on the current data is not justified.

4. Results, p.9, line 12: the phrase "highly evolutionarily conserved" should be replaced by "highly conserved in mammals" since comparisons only included mammals.

5. Discussion, p.14, line 5: Authors refer to a supplementary figure 7 that is absent from the supplementary PDF file and therefore it cannot be reviewed. Is it maybe a typo referring to another figure?

6. Figure 1: The color-codes of panels A and B are not self-explanatory and for clarity, they should be described in the figure legend.

**Authors Response**

**Point-by-point responses to the reviewers’ comments:**

*We would like to thank the reviewer’s for their positive feedback and the insightful comments. We have addressed all point and are convinced that these additional data further increased the quality and the impact of the manuscript. Please find a detailed point-by-point answer below.*

Reviewer #1: Preussner and colleagues present a very interesting and original study describing how skipping of penultimate exons in mammals can extend the C-termini by creating a frame shift that leads to translation into the 3′ UTR. The authors report that at least 10% of human and mouse genes have a penultimate exon that is skipped significantly (as judged by RNA-seq) and that extends the C-termini by at least 20 aminoacids. Then, they show that these extended sequences are enriched for disordered regions and PxxP motifs, which can abolish or rewire protein-protein interactions and lead to decreased protein half-life. The authors suggest this may be a regulatory mechanism for some genes, but also a control mechanism for others, since skipping of penultimate exons does not lead to NMD. Finally, they exemplify their results by focusing on the cases of U2af26 and PDE6G. In general, I believe this is a nice study worth of Genome Biology. However, I have a few comments/concerns:

1) One conceptual issue that requires further clarification and analysis is the very idea of "extended translation into 3′ UTRs upon skipping". It was not clear to me how often this is due to skipping in the canonical isoform, as the manuscript seems to implicitly suggest, or due to skipping of an "alternative", minor, penultimate exon. In other words: is it possible that in many cases they are looking at penultimate exons that are rarely included and, when they are included, they lead to shorter C-termini? I think it would be important to show how often their penultimate exons are part of major or minor isoforms (e.g. by showing the average PSI of the exon). Along these lines, they show the fraction of exons with evidence for skipping by RNA-seq (e.g. ~50% for PSI < 80), but it would be important to also show the fraction of them with significant inclusion (e.g. with PSI > 50 and >90, etc).

*We thank the reviewer for this suggestion. We checked the distribution of mean (PSI_MEAN) or median PSI (PSI_MEDIAN) values across different tissues for the 2152 penultimate exons that are alternatively spliced in at least one tissue. As shown in Fig R1 below, the median values of PSI_MEDIAN and PSI_MEAN are 87.5 and 85.2, respectively. We also checked the distribution of minimum PSI values (PSI_MIN), as well as the distribution of maximum PSI values (PSI_MAX), which shows the fraction of exons with significant inclusion as the reviewer suggested. All these results indicate that most of these penultimate exons are part of major isoforms and they are constitutively expressed in most mouse tissues but spliced out in a few tissues leading to extended translation into 3’UTR.*

*Fig R1: Distribution of minimum (PSI_MIN), median (PSI_MEDIAN), mean (PSI_MEAN) and maximum (PSI_MAX) PSI values across different mouse tissues for penultimate exons that are alternatively spliced in at least one tissue, either for all exons or divided into annotated and novel isoforms.*

*Interestingly, 308 (14.3%) annotated penultimate exons show significantly lower PSI values compared to 1,844 (85.7%) novel ones, indicating most annotated penultimate exons are part of minor isoforms.*

*These results are included in Fig. 1E in the revised manuscript.*

2) Relatedly, it would be interesting to see the numbers for all types of penultimate exons genome-wide. They currently focus on a specific type of penultimate exon, but it is not clear how other penultimate exons behave. Namely: A) penultimate exons that are multiple of three; A.1) penultimate exons that are not multiple of three and lead to shorter C-termini when skipped; and A.2) penultimate exons that are not multiple of three and lead to longer C-termini when skipped (their set of interest). It will be important to compare these three sets in terms of PSIs, conservation, enrichment of PxxP motifs, etc. instead of just comparing their set of penultimate exons to internal exons (e.g. Figure 1E).

*We thank the reviewer for this suggestion. In addition to the 3,233 penultimate exons leading to longer C-termini when skipped (Penulti_long), 87,093 internal frameshift (internal_fs) and 60,007 internal frame-preserving (internal_fp) exons, we also checked 9,791 penultimate exons leading to shorter C-termini when skipped (Penulti_short) and 8,512 exons with length divisible by 3 (Penulti_fp). As shown in Fig R2 below, the skipping of all three kinds of penultimate exons is more frequently observed than that of internal exons. This is especially true when looking for PSIs < 80. As discussed in the manuscript, this might have been a strategy during evolution to experiment with new C-terminal sequences (this also applies to some of the frame preserving exons if they include the canonical stop codon) and skipping of penultimate exons might thus be increased in general. We have included these data as Fig S1F. For the remainder of the manuscript we have focused on the cases with a new C-terminus longer than 20 amino acids.*

*Fig R2: Barplot showing percentage of exons with supporting RNA-seq for internal frame-preserving (Internal_fp), internal frameshifting (Internal_fs), penultimate frame-preserving (Penulti_fp), penultimate frameshifting leading to shorter C-termini (Penulti_short) and penultimate frameshifting leading to longer C-termini (Penulti_long) exon skipping events using different cutoffs for an exon to be considered alternative*

*Similarly, we also checked the distribution of minimum, median, mean and maximum PSI values for Penulti_short and Penulti_fp exons as we did for Penulti_long ones (Fig R3). As the splicing pattern is similar for all three categories of penultimate exons (except more skipping of annotated exons leading to longer C-termini), we have not included these data in the manuscript (but would of course be open to the reviewer’s or editor’s suggestion).*

*Fig R3: The distribution of minimum, median, mean and maximum PSI values across different mouse tissues for Penulti_short (left) and Penulti_fp (right) exons.*

*We also compared the amino acid content before and after skipping for Penulti_short exons (not applicable for Penulti_fp). Fig R4 shows the amino acid length distribution for this category. We only retained 2,862 exons with at least 20 amino acid gained after skipped; these are C-termini that are mainly encoded in an alternative frame of the last exon and do not extend far into the ‘3’UTR’. Fig R5 shows a somewhat similar enrichment of amino acids in these alternative frames; the flexibility is reduced in these cases as the alternative frame does in large parts overlap with the canonical frame in the last exons, which might explain some of the differences when compared with the amino acids in extended C-termini.*

*Fig R4: Length distribution for the amino acid gained after skipping of 9,791 Penulti_short*

*Fig R5: Boxplot comparing the AA content between frameshift and full-length sequences (grey boxes) and between the sequences 100 AA before (CDS) and after canonical stop codons (UTR, white boxes) for 2,862 Penulti_short exons.*

*The conservations for Penulti_short and Penulti_fp exons are shown in Fig R6. 51.1% of Penulti_short exons and 53.0% of Penulti_fp exons are conserved, which is significantly higher than 29.4% of Penulti_long exons. This could indicate that the extended C-termini have developed in a species-specific manner with a species-specific functionality. This however remains speculative at this point. These data are included in the manuscript as Fig. S4D.*

*Fig R6: Schematic illustration of identification of conserved Penulti_short (left) and Penulti_fp (right) exons.*

3) The evolutionary conservation of their penultimate exons based on liftover seems quite low (950 have 1-to-1 orthologous between human and mouse out of 3233 mouse exons, or 29%; Figure 4). This value is typical for alternative exons, but not for "major", highly included exons (see point 1). This is, of course, not a problem per se, and it is still remarkable that the majority of those 1-to-1 orthologs are alternative in both species, but the authors should acknowledge the relatively low level of conservation at the orthologous level and how it compares to the other types of penultimate exons (point 2).

*We thank the reviewer for this suggestion. As shown in the response to question 1), most of these 3,233 penultimate exons are part of major isoform. The relatively low level of conservation could be due to more stringent criteria here. First, not only these penultimate exons are required to have 1-to-1 orthologue between human and mouse, but also the end position of the upstream exon and the start position of the downstream exon. In other words, the upstream and downstream introns of these penultimate exons must have 1-to-1 orthologues. This is to ensure the splice events to be conserved instead of just the alternative exons, where a splice event comprises of upstream exon, alternative exon and downstream exon (please also see response to question 5). Second, only the penultimate exons in both species are considered. For example, if one exon is conserved between the two species, but it is not penultimate exon in one species, this exon is also not considered.*

*Nevertheless, we acknowledge their low level of conservation. Because using the same stringent criteria, 51.1% of Penulti_short exons and 53.0% of Penulti_fp exons are conserved, which are significantly higher than 29.4% of Penulti_long exons (Fig R6). As mentioned above, this may indicate a more species-specific evolution of alternative splicing leading to extended C-termini.*

4)	I think Figures 5F and 5G could be more informative. I agree they show dramatic changes, but it is not obvious what is changing. It would be good to complement it with bar plots for the main interactors in the two isoforms and a few of the "novel" interactors.

*We have altered the representation of the data and also provide individual figures for two specific interaction partners for U2af26 fl and fs.*

5)	Page 15: The authors remove exons that share all start and end positions to avoid multiple counting. However, I do not understand why they count multiple times the same exon if the upstream and downstream exons are different. I think it would be more appropriate to count the actual exons only once, even if they come in different combinations.

*We have considered each exon at splicing-event level, which comprises of upstream exon, alternative exon and downstream exon. The example in Fig R7 shows one alternative exon, but it will be considered twice in the two splicing events. If its skipping leads to extended 3’UTR, both events will be considered as our penultimate exon of interest. If its skipping leads to extended 3’UTR in one event but shorter 3’UTR in the other, only one event will be considered. Splicing-event level analysis could enable us to separate these scenarios.*

*On the other hand, these 3,233 penultimate exons are from 2,860 gene loci. We would be using 88.5% of our current set if we counted the same exons only once. The trends should still hold.*

*Fig R7: A schematic example to illustrate one alternative exon (red) that are considered as two splicing events.*

6)	I have some minor/formal comments about U2AF26. First, its official gene symbol should be provided. Second, assuming it is U2AF1L4, I could not find which was the frame-shifting penultimate exon they are discussing, since the length of the main U2AF1L4's penultimate exon is multiple of three. Looking into their original U2af26 paper, I saw that they might be referring to skipping of the second and third to last exons together. If correct, please acknowledge this, at least in the Figure legend. Otherwise, perhaps clarify the issue.

*Both points are correct. It is U2AF1L4 and it is skipping of exons 6 and 7 that lead to the alternative frame. We have clarified this in the revised version and thank the reviewer for pointing it out.*

Reviewer #2:

In the current manuscript, Preussner et al are providing an evidence for the coding potential of 3' UTR in human cells. Using the computational approach, they predict skipping of the penultimate exons by alternative splicing that leads to the frame shift and extension of mRNA coding potential in the areas of 3' UTR beyond the translation termination stop codon. Such events can stay undetected by the nonsense mediated decay machinery and lead to the production of the proteins with novel C-termini that differ from currently annotated isoforms. Authors have validated the presence of the peptides that can arise in such a manner using mass spectrometry of the mouse brain tissue. This is an exciting observation, since such a peptides are frequently overlooked/masked during the mass spectrometry experiments. Amino acid sequence analysis of "translate through" proteins is enriched in proline-rich motifs, both in mouse and human samples, which suggests their role in mediating protein-protein interaction.

Expression of 3' UTR derived portions of protein sequences fused with GFP exposed their reduced stability, likely through increased proteasome degradation rate. In addition, changes in C-terminal sequence results in the rewiring of the protein-protein interaction network for some of the investigated examples (like U2AF26) which can have a potential downstream effect on the protein targets.

Using CRIPSR/Cas9 authors have recapitulated a previously reported mutation in the exon 3 of PDE6G that leads to the usage of cryptic 5' splice site and "translate through" canonical stop codon. This alteration does not affect the expression level of the mutant allele on the RNA level that stayed comparable to the wild type. At the same time, however, the drastic effect is observed on the protein level. The amount of protein appears greatly reduced potentially due to the destabilization by proteasome. Co-immunoprecipitation of the C'- term. extended protein suggests a complete loss of the interaction with another subunits of the complex.

Mechanism proposed here points towards the idea of unstable protein production due to the high proteasome degradation rate of PDE6G. Homozygous mutation observed in patients in this scenario would lead to almost complete loss of the functional and stable PDE6G that is a necessary component of the protein complex, promoting rapid and early retinal degeneration. I believe this work would be interested to the wide audience of biologists

I have several comments regarding the current work:

1.	Brain tissue is the most enriched in the alternative splicing events that drastically and dynamically change throughout the development, is it possible that the high detection rate of extended proteins in this tissue is simply correlated with the high alternative splicing activity and large amount of alternatively spliced exons?

*This is an interesting point. We have compared the number of penultimate exons where skipping leads to an extended C-terminus in different tissues (Fig. S1B). There is basically no difference between the different tissues in the number of these splicing events. It thus appears that this subgroup of alternative splicing events is not more prevalent in brain (as are other alternative splicing events). We thus believe that the frequency of the alternative splicing events does to induce a tissue-specific bias towards higher detection of the respective protein isoforms.*

2.	Despite relatively high observation rate (10% reported here) of the 3' UTR translation the evidence of the molecular and biological functionality is still vague. High degradation rates, reduced high-life time is suggestive of the quality control (fail safe) mechanisms. Would be great to add this to the discussion and dial down the functional role of the 3' UTR coding.

*We have sharpened the discussion to make this point clearer. The first paragraph of the discussion now reads:*

*We find two features of these extended C-terminal sequences: a) they show a significantly higher frequency of protein-protein interaction motifs, which may contribute to rewiring of protein-protein interaction networks, and b) in many cases they act in a destabilizing manner (Figure 7). We suggest that the latter is due primarily to elevated content of proline residues leading to higher levels of protein disorder. As the respective mRNAs are not recognized by NMD this could represent a surveillance pathway to correct splicing errors, but it could also contribute to control protein levels in a regulated, e.g. tissue-specific, manner.*

*Both mechanisms are discussed in more detail in additional sections of the discussion.*

3.	Did you find examples where the C' terminal extension encoded for a known protein domain or at least parts of it?

*We have looked for a global change in known protein domains. Consistent with a higher level of disorder, we find less known domains in the fs vs the fl C-termini (Fig R8). As these data confirm a finding that is in the manuscript (higher disorder), we have not included the additional data.*

*Fig R8: Comparing the structured domains in frameshift (fs) and full-length (fl) isoforms using the CD search tool focusing on mouse isoforms confirmed by RNA sequencing.*

4.	Do these rather unstructured and flexible proteins parts affect the phase separation properties? Would be great to predict it computationally using some of the recently published tools. For example with: https://doi.org/10.1101/842336, catGRANULE or any others.

*This is also an interesting suggestion. We have used catGRANULE to compare the phase separation behavior of frameshift (fs) and full-length (fl) isoforms (focusing on mouse isoforms confirmed by RNA sequencing). This reveals no global difference in the phase separation behavior (Fig R9; fs mean: -1.667; fl mean: -1.568). Interestingly, the phase separation score for fs and fl C-termini does not correlate (R2 = 0.001) indicating different behaviors of the two C-termini in individual cases. An interesting example is the transcription factor Taf15 (which is known to form polymeric fibers; Kwon et al., Cell, 2013): Here the fl isoform has a high propensity to phase separate (score 9.4) while the frameshifted C-terminus has not (score - 1.3). We have not included these data in the manuscript as it leads somewhat away from the main points, but are open to suggestions.*

*Fig R9: Phase separation propensity of frameshift (fs) and full-length (fl) isoforms focusing on mouse isoforms confirmed by RNA sequencing (n=2152).*

5.	What are the go-terms associated with the identified the mRNAs that have 3' UTR coding potential? For instance, AS in neurons was recently shown to be enriched in genes coding for ion channels. In addition, AS exons can be regulated by neuronal activity itself (Gonatopoulos-Pournatzis T., Mol. Cell 2020).

*We thank the reviewer for this suggestion. GO enrichment analysis was performed for the mouse genes with alternative penultimate exons in each tissue, as well as all tissues and in conserved events (mouse and human, independent of tissue) using Enrichr (*[*https://amp.pharm.mssm.edu/Enrichr*](https://amp.pharm.mssm.edu/Enrichr)*).*

*While some GO terms are enriched for individual tissues, we have focused on events that are conserved between human and mouse and show these GO terms in Fig. S4C.*

6. The regulation of AS in the CNS is relatively well characterized and key regulators of multiple alternative exons are known. These RBPs also exhibit a dynamic expression pattern at different stage of neuron development and maturation (Weyn-Vanhentenryck, 2018, Nature Com). Is there an enrichment for certain RBP targets in the catalog of mRNAs described here that are associated with penultimate exon skipping? This can help to understand if such a skipping is an aberrant event or rather functionally regulated mechanism.

*We thank the reviewer for this suggestion. To check an enrichment for certain RBP targets in penultimate exons, we used the neuron differentiation dataset to illustrate this. Out of 3,233 penultimate exons, 1,635 are expressed in all differentiation stages, which are further considered for motif analysis to exclude gene expression effect. We selected as BACKGROND 895 exons that are constitutively spliced in (PSI>90) or constitutively spliced out (PSI<20) in every sample. We also selected as TARGET 276 exons that show differential splicing pattern between any two stages (ΔPSI>20) for RBP binding motif enrichment analysis. To do this, we used the sequences of these penultimate exons as well as their 50bp upstream and downstream introns as input for Homer with the following command:*

*findMotifs.pl TARGET.fa fasta OUTPUT -fastaBg BACKGROUND.fa -rna -len 6,7,8*

*Then we retained the motifs with 1) p-value < 1e-5; 2) frequency in TARGET > 15%; and frequency in BACKGROUND < 5% to match the motifs annotated in RBPmap database (http://rbpmap.technion.ac.il/index.html). None of the motifs show significant enrichment for the 276 exons.*

*We also tried a more stringent cutoff – ΔPSI>50 to define differentially spliced exons. Despite of the smaller set (72 exons), we found two significant motifs using the same background and the same method, which match Pcbp3 and Mbnl1, respectively (Fig R10).*

*Fig R10: Two motifs that are significantly enriched in the penultimate exons showing differential splicing pattern across neuron differentiation stages (ΔPSI>50 between any two stages). They match to Pcbp3 motif and Mbnl1 motif, respectively (de novo detected motifs are shown on top and annotated motifs are shown on bottom).*

*Interestingly, both genes show neuron differentiation stage specific expression pattern (Fig R11), which indicates a concerted regulatory mechanism and potentially functionality of these penultimate exons. These data are included in the revised manuscript as Fig S1G, H along with a citation of the Weyn-Vanhentenryck et al paper.*

*Fig R11: Barplot showing gene expression across neuron differentiation stages for two splicing factors Pcbp3 (left) and Mbnl1 (right)*

7. Is G/T a common mutation in RP? Can be mentioned in the discussion.

*The G/T mutation is one of many mutations leading to RP and this particular mutation is a rare mutation.*

Minor comments:

1. What is the protein expressed in the control lanes as a FLAG fusion in figure 6E. Needs to be better described in the figure legend.

*We have used the N-terminus of the unrelated RNA helicase Brr2 as Flag-tagged control protein with similar expression level. This is now also mentioned in the figure legend.*

2. Also, the co-IP's (figure 5F and G) are not presented in a visually appealing way, which makes them less intuitive. Please modify and label the top protein partners that have been lost/gained due to the C-terminus modification.

*We have modified the figure according to the reviewer’s suggestion.*

Reviewer #3:

In this work, Preussner and colleagues present a thorough report of a conserved expansion of the protein-coding potential of 3΄UTRs in mouse and human cells. Following a bioinformatic analysis that reveals alternative splicing as a common mechanism to extend ORFs in the 3΄UTRs, they observe and biochemically characterize an increase of C-terminally-extended proline-rich motifs that can reduce the protein stability of the corresponding isoforms and in the case of U2af26 they show a profound impact on its interactome. Last, they relate this phenomenon with a retinitis pigmentosa-causing mutation, highlighting the clinical significance of these findings. Overall, this is a work of broad significant interest, the experiments are conducted carefully and convincingly to provide a thorough characterization of this phenomenon and the manuscript is easy to follow. After addressing the points below, I judge this manuscript suitable for publication.

Major points

1. Results, p. 9: Concerning the verification of expression of the 3΄UTR-encoded variants on protein level: Is there a reason why a mass spectrometry analysis was not also performed in human cells? It would be advisable to verify the ORF extension in the 3΄UTR in the same way as in mice. This would strengthen the biological relevance of the phenomenon and would allow a more direct comparison with the results from mouse cells.

*This is an interesting point. For our analysis we wanted to use primary tissue, which has to be fresh and quickly snap-frozen to prevent degradation of the unstable fs proteins. Therefore we have used mouse primary tissue. In the past weeks we were not able to perform our own experiments with human material and therefore had to rely on published studies. We used a dataset from Geiger et al., 2012 (Molecular and Cellular Proteomics), which contains mass spec experiments from 11 human cell lines and searched against our custom-made library including human fs C-termini. We find a total of 59 peptides that uniquely map to C-terminal sequences. However, 49 of them were already in the UniProt database, reported as ‘isoforms’ (this may not be too surprising, as there are many more isoform entries for the human database). This analysis clearly shows that fs C-termini are also expressed at the protein level in humans. On the other hand, the number is lower than what we observe in mouse brain. This could be due to different preparation of proteins and peptides, different settings during the actual measurement, or differences in the tissue type. However, it is evident that sequences that are accessible through frameshift-inducing alternative splicing of penultimate exons are translated in mouse and humans. Due to the differences between our data and the Geiger et al. data mentioned above, we would not include the human data in our manuscript, but we are of course happy to take the reviewer’s or editor’s advice on this matter.*

Additional points

1. Results, p.5, lines 5-12: Authors convey the notion that protein-coding genes are not expected to contain untranslated exons in their 3΄UTRs. Introns in the 3΄UTR of a gene are indeed expected to stimulate NMD through the deposition of an exon-junction complex (EJC) during splicing of the corresponding mRNA. However, even though a splicing event in the 3΄UTR is expected to trigger NMD, this does not mean that the entire population of this mRNA is rapidly degraded. The sensitivity of a transcript population to NMD varies, depending on several factors (number of splice junctions and length of the 3΄UTR, the presence of uORFs, etc.) and there are examples of protein-coding mRNAs with a termination codon in penultimate exons (such as human CALM3 - calmodulin 3 mRNA). I suggest editing this segment accordingly to consider this point.

*We have rephrased this paragraph and included the possibility of splicing in the 3’UTR as an additional mechanism to induce NMD.*

2.	Results, p.6, lines 1-2: Since the results of this work show that extending the coding potential of the 3΄UTR is conserved it could be that some of these protein isoforms may have biological significance and therefore are not necessarily degraded from the cell. Taking this, the sentence of p.8 lines, 32-33 and parts of the discussion (e.g. p.13, lines 24-25) into consideration, I propose rephrasing this sentence accordingly.

*We thank the reviewer for pointing this out and agree, that the sentence on page 6 was too much focused on NMD. We have simply deleted this sentence and think that it is now clearer that we suggest a dual role of extended C-termini: either functional rewiring of protein interactions or control of protein stability.*

3.	Results, p.6, lines 5-7: To address this question, direct evidence on the bulk translation of these mRNAs would need to be provided (e.g. Ribosome profiling evidence). Therefore, a broader re-definition of 3΄UTRs based on the current data is not justified.

*We have deleted the respective sentence.*

4.	Results, p.9, line 12: the phrase "highly evolutionarily conserved" should be replaced by "highly conserved in mammals" since comparisons only included mammals.

*This has been corrected.*

5.	Discussion, p.14, line 5: Authors refer to a supplementary figure 7 that is absent from the supplementary PDF file and therefore it cannot be reviewed. Is it maybe a typo referring to another figure?

*We apologize for the missing figure. There should have been a summary figure attached as figure 7. It is included in the revision version.*

6.	Figure 1: The color-codes of panels A and B are not self-explanatory and for clarity, they should be described in the figure legend.

*We have updated the figure legend.*

**Second round of review**

**Reviewer 1**

The authors have successfully addressed all my comments and concerns. I congratulate the authors on their work.

**Reviewer 2**

This is a revised manuscript by Preussner et al examining the effect of alternative splicing on coding potential of mRNA. Even though a proof for the functionality of the novel and unstructured C termini is somewhat incomplete, this work is opening an exciting discussion about the role of penultimate exons and 3’ UTR translation. Current study will be interesting for the audience of Genome Biology and I see no further obstacles for publishing it.
